# Risk Factors and Consequences of Food Neophobia and Pickiness in Children and Adolescents: A Systematic Review

**DOI:** 10.3390/foods14010069

**Published:** 2024-12-30

**Authors:** Carmen del Campo, Cristina Bouzas, Josep A. Tur

**Affiliations:** 1 Research Group in Community Nutrition and Oxidative Stress, University of the Balearic Islands-IUNICS, 07122 Palma de Mallorca, Spain; farocreus@yahoo.es (C.d.C.); cristina.bouzas@uib.es (C.B.); 2 CIBER Fisiopatología de la Obesidad y Nutrición (CIBEROBN), Instituto de Salud Carlos III (ISCIII), 28029 Madrid, Spain; 3 Health Research Institute of Balearic Islands (IdISBa), 07120 Palma de Mallorca, Spain

**Keywords:** pediatric food neophobia, pickiness, risk factors for picky eating, children

## Abstract

Food neophobia and pickiness are the resistance or refusal to eat and/or avoid trying new foods due to a strong reaction of fear towards the food or an entire group of foods. This systematic review aims to assess evidence on the risk factors and effects of food neophobia and picky eating in children and adolescents, giving elements to avoid the lack of some foods that can cause nutritional deficiencies, leading to future pathologies when they are adults. A systematic literature search was performed in Medlars Online International Literature (MEDLINE) via Pubmed and EBSCOhost, LILACS and IBECS via Virtual Health Library (VHL), Scopus, and Google Scholar. MeSH terms used were: ((food neophobia [Title/Abstract]) OR (picky eating [Title/Abstract]) OR (food selectivity [Title/Abstract])) NOT ((anorexia nervosa [MeSH Terms]) OR (bariatric surgery [MeSH Terms]) OR (avoidant restrictive food intake disorder [MeSH Terms]) OR (autism spectrum disorder [MeSH Terms])). One hundred and forty-two (n = 142) articles were selected for children and adolescents (0–18 years old). They were structured according to contents: prevalence, risk factors, consequences, strategies and treatment. The studies showed a prevalence of the need for intervention on modifiable risk factors. Food neophobia and pickiness developed in childhood are conditioned by risk factors related to biological, social, and environmental characteristics, as well as family education and skills. Strategies to minimize or avoid these disorders should be aimed at implementing healthy habits at these levels.

## 1. Introduction

Food selectivity, neophobia, aversion, and avoidance, as well as picky eating, are all terms used to describe that children do not eat [1]. In children and adolescents, the concept of food neophobia is linked to eating or appetite disorders, picky eating or food fussiness [2]. Eating disorders are defined as those with some of the following characteristics in the individual way of eating: not eating enough, eating frequently very selectively, eating frequently slowly, and showing unwillingness to eat certain foods [3].

Food neophobia and fussy eating behavior are determined by biological, anthropological, economic, psychological, socio-cultural, and home-related factors, and their influences compete, reinforce, and interact with each other [4]. This behavior is one of those responsible for a decline in the variety of food consumption and, conversely, of nutritional intake, as well as be a barrier to ensuring that the child eats healthy diets, associated with a high level of conflict and stress in the family and school environment at meal time [5]. Fussy eating behavior is a significant challenge for many families of school-aged children. Parents’ feeding goals, practices and the negative emotional impact of fussy eating have been widely reported in previous studies [6,7,8]. It has been pointed out how a mother reported little concern despite her son eating little amount of vegetables, or he did not eat vegetables at all; other parents reported higher levels of concern, uncertainty and inconsistent practices despite their child reportedly consuming a relatively balanced diet; many parents were aware of strategies for managing picky eating challenges, but encounter difficulties implementing these strategies; other parent restricted their children from eating sweet foods in the past, but as the child grows up and goes to school, educating the child to be able to make their own choices becomes a higher priority; and other parents avoided conflicts and provided a balanced meal, or wanted a child to eat more and promoting child autonomy/self-regulation. Most parents adapted their goals in front of the children’s picky eating according to contextual factors such as time, energy levels and day of the week; many parents felt it important to create a positive mealtime environment and expressed efforts to avoid conflict [5].

Food neophobia or aversion is defined as the resistance or refusal to eat and/or avoid trying new and unfamiliar foods due to a strong reaction of disgust or fear towards the food or entire group of foods, both visually and tasty [9]. Sensory characteristics have been reported as the most influential determinants of eating behavior, and among these, textures are the main reason for food rejection or acceptance in children [10,11,12]. The feeding style (urging the child to eat, unpleasant emotions during the meal, parental eating habits, neophobia in the mother, and others) has also been reported as having a great effect on the food neophobia appearance [13]. Food neophobia is one of the children’s eating behaviors that causes parents great concern [14,15]; it has been included in the group of feeding disorders or selective eating, which is part of the broader group of sensory food aversions [16]. Neophobic behaviors can appear as early as the first year of life but most often intensify between 18 and 24 months of age, which is related to the child’s increased mobility. Food neophobia is a natural development stage that usually resolves spontaneously, but its occurrence can influence the perpetuation of future inappropriate behaviors [1].

Picky eating is generally classified as feeding disorder, and it has been defined as unwillingness to both eat familiar foods and try new foods, and showing strong food preferences severe enough to interfere with daily routines to the extent that is problematic to the parent, child, or parent–child relationship [17], as well as restricted intake of food, leading inducing parents to provide menus different from those of the other family members [18]; a low number of food in the diet, special preparation of foods required [19,20] and consumption of inappropriate food variety by avoiding/rejection of familiar or unfamiliar foods [21,22]. The age of picky eating in children has been reported below the age of 1 year, perhaps related to parental picky eating [17], and it mainly appears in populations from developed countries despite their ethnic origin [23,24]. The picky eating development can be affected by the following factors: parental feeding style and monitoring, pressure to eat, personality, social influences [23,25], reduced duration of breastfeeding, and early introduction of complementary foods [26,27].

The neophobic attitude is evolutionary and important to protect individuals from ingesting dangerous foods; as omnivorous species, to survive, humans have to distinguish between safe and poisonous food [1]. This dislike for one or more foods could be considered an adaptive and emotional reaction of human beings to protect themselves from contamination or disease and feel safe in front of plants or animals with toxic properties [28]. Currently, neophobia is a useful mechanism in early childhood linked to the development of a sense of taste; the attitude of distrust toward novelty protects the child from the danger of eating something potentially dangerous to health [29]. Hence, these avoidance behaviors can also be beneficial for obtaining and consuming food [1].

Food preferences and aversions are shaped by the chemosensory system underlies taste, touch, sight, and smell perception [30]. Consequently, disgust in front of foods motivates avoidance behavior and triggers specific disgust stimuli, which are responded to through parasympathetic activation, activation of specific facial muscles, appraisals of contamination, and oral rejection [20].

The main differences between food neophobia and pickiness are that food neophobia is usually considered as the refusal to eat/avoid new foods and to refuse to eat unknown foods. Instead, picky eaters are generally defined as those children who consume an inappropriate variety of foods, with a lack of will to eat certain foods that are meticulous, by rejecting a high number of familiar and unfamiliar foods, both novel and traditional to them. It appears that the picky eaters will need more exposure to certain foods to accept them than the food neophobes [21,31].

Pickiness and food neophobia are closely related in terms of some characteristics of the individuals. In some cases, both disorders coexist [31]. Food neophobia and pickiness showed that inter-relationships and social and parental factors would have analogous effects on the magnitude and length of expression in both food neophobia and pickiness. These parameters can be modified in different ways by age, taste/tactile ability, culture, and environment [21]. Food neophobia is part but not the entirety of pickiness behavior and a constituent of unwillingness to try novel foods. The effect of food neophobia on the child’s willingness to try a new food decreases from the first taste assimilated as positive; after that, the rejection of a flavor will no longer be part of the child’s food neophobia, and persistent rejection should be considered as part of pickiness. Developmental aspects of food neophobia and pickiness are linked to the child’s mobility, resulting in a slower period of maturation in pickiness than in food neophobia [21].

Rejection of several foods, mainly vegetables and fruits, may cause serious health consequences due to deficiencies of certain essential nutrients, especially vitamins and minerals, derived from the absence of these foods in the diet [1,32]. Neophobic children eat fewer vegetables, fruit, milk, and dairy products than those recommended, with negative consequences on health resulting from a poor diet [33,34]. All of these food avoidances may be especially harmful to children and adolescents since they are growing organisms.

To establish the health strategies needed to modify eating patterns, it was necessary to create useful tools to measure the level of food neophobia, as well as to determine which foods are necessary to define the most appropriate interventions. Currently, there are validated and established tools and scales as references widely used by the scientific community. Some of these tools were validated in different populations due to the differences found in neophobias linked to the food characteristics and culture of different ethnic and/or socioeconomic groups [35].

The aim of this systematic review was to evaluate the evidence on the risk factors and effects of food neophobia and picky eating in children and adolescents, giving elements to avoid the lack of some foods that can cause nutritional deficiencies, which will lead to future pathologies when they are adults.

## 2. Methods

### 2.1. Review Protocol, Information Sources, and Search Strategy

The Preferred Reporting Items for Systematic Reviews and Meta-Analyses (PRISMA) [36] guidance was used, with the protocol of this systematic review registered in the international prospective register of systematic reviews (PROSPERO ID: CRD42024566754). This systematic review also followed the PICOS (Population, Intervention, Comparison Outcomes, and Study type) guidelines for systematic reviews commonly used to identify components of clinical evidence for systematic reviews in evidence-based medicine [37,38]. PICOS used are shown in Table 1.

The systematic review was performed up to April 2024. Searched literature has been retrieved from the MEDLINE database via PubMed and EBSCOhost, LILACS and IBECS via Virtual Health Library (VHL), Scopus, and Google Scholar, using the following combination of Medical Subject Headings (MeSH) terms: food neophobia [Title/Abstract] OR picky eating [Title/Abstract] OR food selectivity [Title/Abstract] NOT anorexia nervosa [MeSH Terms] OR bariatric surgery [MeSH Terms] OR avoidant restrictive food intake disorder [MeSH Terms] OR autism spectrum disorder [MeSH Terms]. Seven hundred and thirty-four articles were initially selected.

### 2.2. Inclusion Criteria

Original peer-reviewed research papers written in English or Spanish, only in humans, and made between 2000 and 2024 were considered. A total of 479 articles were obtained.

### 2.3. Exclusion Criteria

Reviews and case reports were excluded. Other exclusion criteria were irrelevant study objectives for the current review, irrelevant study design for the review and irrelevant association. Studies where the measurement of food neophobia was made on a population with diseases (cancer, intolerance, allergies, etc.) and patients with ARFID (Avoidant/Restrictive Food Intake Disorder), were excluded since it is an eating disorder in the food fussiness domain, causing more severe impairments than food neophobia and limiting a wider range of food intake [39]. After that, three hundred and twenty-seven articles were selected. The last exclusion criterion applied was the age of the population (0–18 years old), discarding those carried out on adults (≥18 years, n = 95), mixed ones (0–99 years, n = 2) and those carried out on children with content irrelevant to the study (n = 90). Finally, 142 articles conducted in the pediatric population (between 0 and 18 years) were selected.

**Figure 1:**
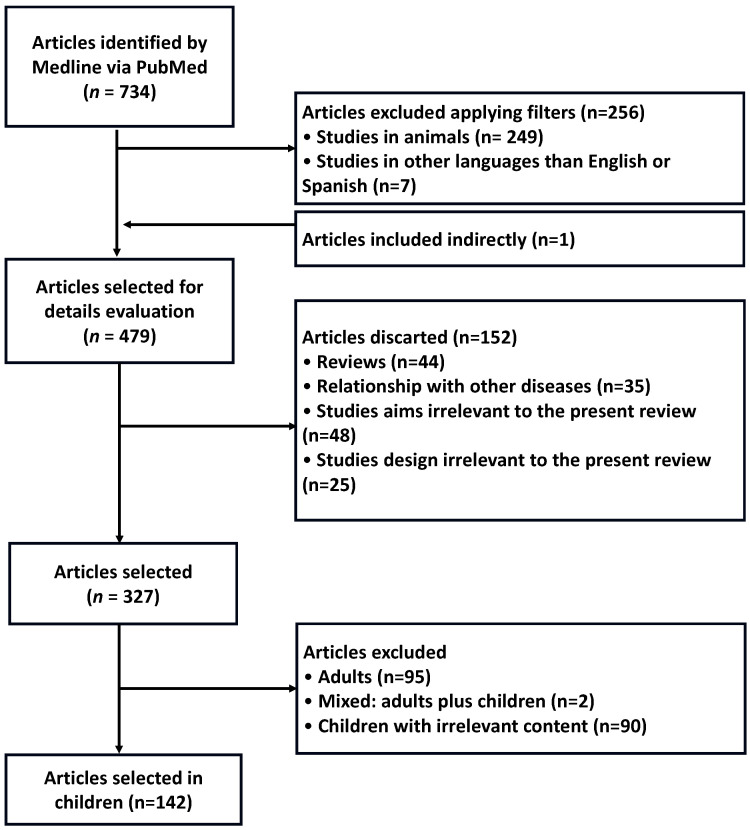
Flowchart highlighting the selection process of articles.

A flowchart to report on the information flow during different stages of this review, showing the number of literature records found, included, and excluded, as well as the rationale for exclusion, has been created and shown in Figure 1.

### 2.4. Study Selection

Initially, titles and abstracts of papers were screened for the relevance of their thematic fit, related to the focus or theme of the study. Following title and abstract screening, full texts of selected studies are thoroughly examined in the next step to assess their eligibility. If the information in titles and abstracts was unclear, additional context or full-text examination was used to make an informed decision. The articles were reviewed by at least two reviewers and were considered for the selection criteria listed by the Joanna Briggs Institute, a procedure to independently assess the methodological quality of scientific articles [40]. Two reviewers performed screening independently. Any discrepancies or disagreements in the screening process were addressed through discussion and consensus among the reviewers and the third author if needed.

### 2.5. Data Collection and Extraction

Two team members extracted data independently, which was critically reviewed by the other team members at the end of this step. Articles were classified into new tools to measure neophobia; classification and prevalence of food disorders and related risks; effects of food neophobia and picky eating risk factors; consequences linked to food neophobia; macronutrient and food restriction; micronutrient restriction; food neophobia strategies and treatment; other minor results.

The quality of analyzed studies, including the risk of bias, was assessed through the Cochrane Risk of Bias-2 tool [41], as well as the Newcastle–Ottawa Scale [42] for non-randomized studies, including case-control and cohort studies, although some criticism of it has been reported [43].

## 3. Results

For the PICOS, 44.5% of articles were relevant after the title and abstract stage (327 articles/734 articles), and 43.4% (142/327 articles) were confirmed to meet the inclusion criteria after full-text review.

The 142 selected articles focused on the prevalence, risk factors and consequences, and strategies to minimize or avoid food neophobia and picky eating in children. Specific measurement tools were used to determine these questions, detailed in Table 1. Eighty-seven percent of the articles reported results in equal proportion on the terms food neophobia (FN) and picky eating (PE); 13% of the articles did not directly determine the concepts neophobia or pickiness, but their results were in line with feeding difficulties in childhood, matching the determinant causes and risk factors.

The results were obtained by comparing the neophobic and neophilic groups or PE with non-PE or by comparing the distinct levels of neophobia and pickiness, respectively. According to the age of the individuals in the sample, the information under study was obtained through surveys carried out on the individuals themselves (self-reported) and sometimes obtained from parents’ and caregivers’ responses (indirect information), with the consequent bias that the first option may entail.

The scales or measurement tools used in these studies were mainly: the Child Food Neophobia Scale (CFNS; n = 34), Food Neophobia Score (FNS; n = 31) and Children’s Eating Behavior Questionnaire (CEBQ; n = 26). Validated but minority measurement tools were used in 22 articles. The methodology was used in 28 articles without validation and only with observational measures. Among them, eight of these articles [44,45,46,47,48,49,50,51] talked about the validation of new tools to measure neophobia under different premises, such as adaptation to different origins of the population or delimiting the FN or PE measured to specific food groups.

One hundred and six articles were cross-sectional, 17 were prospective, 10 were Randomized Controlled Trials, and 3 were cases and controls. Minority studies (n = 4) were cohort (n = 1) and clinical trials.

A low risk of bias in the reviewed articles was determined by applying the domains of the Cochrane Risk of Bias-2 tool (Domain 1: Risk of bias arising from the randomization process; Domain 2: Risk of bias due to deviations from the intended interventions; Domain 2: Risk of bias due to deviations from the intended interventions; Domain 3: Missing outcome data; Domain 4: Risk of bias in measurement of the outcome; Domain 5: Risk of bias in selection of the reported result; and Overall risk of bias). The quality of case-control and cohort studies, following the Newcastle–Ottawa Score, was high (total quality score = 7).

The sample size was very different between the reviewed papers. Most of the papers followed from 100 to 500 subjects. Just 28 studies were carried out on populations with less than 100 individuals, and 19 studies followed more than 1000 individuals. The highest studies were developed in Bristol (UK) as a prospective study on 6608 children aged 4–15 months [35] and in Avon (UK) as a longitudinal study on 7285 children aged 7 years [52].

All studies assessed the 0–18-year-old pediatric population, except two papers that worked on 19–20-year-old or older subjects [53,54]. Studies were conducted on the USA, European, Asian, and Australian populations. All findings are shown in Table 2.

### 3.1. Results on Prevalence of Food Disorders and Related Risks

Seventeen papers assessed the classification and prevalence of food disorders, as well as the related risks for serious disorders, such as FN, PE or even Avoidant Restrictive Food Intake Disorder (ARFID). Bialek-Dratwa et al. [55] classified children into four groups, from risk level 0 (no risk) to level 3 (highest serious food disorders). In a cross-sectional study on 2550 children [56], picky eating disorders were classified into two groups: persistent and non-persistent, with a prevalence of 8 and 5%, respectively. Other studies classified children as slightly persistent, medium persistent, and somewhat persistent [57]. In a prospective study conducted on 2068 children in 2022, a PE prevalence of 16.7% was reported [58]. Another prospective study [59] showed a prevalence of 22.5% for persistent pickiness. The prevalence of PE in cross-sectional studies was 67.5% [60], 29% low persistent PE, 57% medium persistent and 14% high persistent PE [61], 34% PE [57], 30% severe PE [62], 26% PE [63], 4.9% severe PE [44], 62% PE [64], 25.1% PE [65], 59.3% PE [66], and 54% PE [67].

The prevalence of food neophobia has been quantified around 14–16% [57,68,69], but other studies reported a much lower prevalence of 1.8% [70].

Associations have been described between children’s PE and parental feeding habits or mother feeding habits, but no sex effect as father–boy and mother–girl associations have been described in the reviewed papers, except one article describing how mothers influenced fruit and vegetable consumption of daughters through their own patterns of fruit and vegetable consumption and influencing tendencies of daughters to be picky [71].

### 3.2. Effects of Food Neophobia and Picky Eating Risk Factors

Among the articles reviewed, 48 studies dealt with the effects of risk factors that can cause or amplify neophobia and food selectivity or picky eating (PE), including genetic and, environmental and social factors [72,73,74]. Regarding genetic factors, the heritability of being picky and neophobic is a highly transmissible trait [75], up to 72% [76]. There were studies on environmental factors with very different results. Food neophobia was pointed out around 14–16% [57,68,69], but other studies reported a much lower prevalence of around 1.8% [70].

Regarding age as a risk factor, it was pointed out that a child’s age determines the extent to which several factors affect food picky eating [77], as well as the point of highest level of pickiness appears at 4–5 years of age [22,50,78]. It was also determined that the age of onset was 3–4 years [79]. Several authors reported a decrease in the persistence of pickiness with age (56,80,81), which remained stable from preschool to school age [67]. In the Donald study, a decrease in neophobia during adolescence was also described [82].

Analyzing sex as a risk factor, it was shown that there was higher risk of developing picky eating in males [22]. No relationship was observed between sex and FN [69,83], but females showed differences in the intake of vegetables according to the FN category [84]. There is no association between PE and ethnic or maternal BMI [81], except one study showing higher risk of picky eating in children of obese mothers [85].

The risk factors with the highest influence on the development of picky eating and food neophobia were related to parental strategies, showing a direct effect [52,86,87] from the parents’ habits, education, feeding (maternal or external breastfeeding, and introduction of solid foods), the form of exposure, and socioeconomic factors surrounding the family nucleus.

Breastfeeding and its duration were declared a conclusive risk factor in the FN and PE development [88]. There was evidence that exclusive breastfeeding until 4 or 5 months compared to exclusive breastfeeding only from 0 to 1 month decreased the likelihood of pickiness [89]. The late introduction of solid and semi-solid foods into the child’s diet was associated with higher levels of picky eating [32] and FN [27]. However, other studies showed that the age of introduction of solids did not show an association with the dietary risk posed by picky eating and FN [90].

The main difficulties at feeding time were the perceived tension at mealtime, both by the children themselves and externally [6,64,91], parental anxiety [79,92], and those derived from an increased pickiness [93] and neophobia [94]. Persistent PE can be a symptom/sign of pervasive developmental problems but does not predict other behavioral disorders. Remitting PE was not associated with adverse outcomes of mental health [73].

Several studies found that there was an increased risk of developing picky eating when parents were picky eaters [62,95]. Mostly, parents and caregivers themselves were unaware of the relationship between their neophobias and those developed by the children [96], with a positive association between children’s neophobia and those of parents [82,97,98]. Neophobic mothers usually exposed their children to unhealthy foods and consumed less fruit and vegetables, and their neophobias usually coincided with those of their children [26,99,100]; therefore, they can be considered as predictors of food behavior in children [101]. On the other side, mothers who are high consumers of fruits and vegetables usually put less pressure on their daughters, who are less demanding of food [71]. An isolated study showed that knowledge of vegetables, identification of sensations, and the willingness to try vegetables had no effects on neophobia [102].

The kind of education and family eating practices had a direct implication on neophobia and picky eating development [103,104], and parental neophobia is a food fussiness independent determinant [77]; however, further research would be needed to determine it [105]. So, promoting autonomy and praise had a positive influence on the consumption of vegetables [106], as well as a positive climate before mealtime [107]. On the other hand, control and coercive eating practices increased PE levels [104] and led to more problematic behaviors [108]. However, this review found a study that showed the opposite; it said that the covert control of mothers improved the quality of the children’s diet [109].

The parental socioeconomic status was not associated with picky eating [48], but several articles showed an association between picky behavior and low income [110]. Home food insecurity is precisely another factor to be considered because it can make it difficult to access and be exposed to foods such as fruits [111,112]. Moreover, higher neophobia levels in rural areas were due to lower exposure to different foods [103] compared to urban areas.

Regarding relationships with senses, FN was slightly correlated with high taste and olfactory reactivity in children, which could trigger low consumption of fruits and vegetables [112,113,114]. On the other side, FN was not related to tactile enjoyment [115], but children who were defensive to touch had more picky traits [116]. The food’s visual appearance also played an important role in FN [117]. It can be concluded that low-sensitive individuals showed lower levels of food neophobia and higher food acceptability [118].

### 3.3. Results on the Consequences Linked to Food Neophobia

There was no significant association between FN and altered BMI [24]. However, nine studies showed that medium and high persistence of pickiness was associated with low BMI [32,61,119,120] and slightly lower weight [58,67,114,121,122]. It also showed the decreasing effect of pickiness on fat mass [119], but at the same time, with no effect on body fat percentage and fat mass index [123]. It was also found lower BMI in children of parents worried because their children did not eat and parents who pressured children to eat [124]. Few articles reported that food neophobia predisposed to overweight [125] or that obesity and overweight were significantly higher in food neophobic and picky eaters [126].

No significant differences were reported between the dietary intake of PE and non-PE [127], but differences both in behavior and food consumption [2] and total energy intake [128] were also found. The most important consequence was the association of food neophobia with poor diet quality [69,129], due in some cases to the lack of interest in food that neophobia induces [130]. High levels of FN and picky eating were associated with lower hedonic reactions to food [19], and strongly relationship with fruit and vegetable consumption [104]. High adherence to the Mediterranean Diet was associated with a low level of food neophobia and better hedonic scores regarding food [8]. An association between high levels of food neophobia and low awareness of hunger and satiety in childhood was also reported [103]. An FN increase appeared with some textures [131].

### 3.4. Macronutrient and Food Restriction

Picky eaters consumed sugary foods and drinks more frequently [132,133] and low fruits, vegetables [101,120,128,131], and proteins [67,81,134,135]. High levels of neophobia were also related with low fruit and vegetable intake [70,84,125,136,137,138,139], and a reduced intention to try them [99,140,141], high intake of saturated fats [33] and, specifically, of trans fatty acids [85]. Moreover, low fruit and vegetable consumption and decreased protein intake were reported among sedentary people living in suburban areas [82]. Low levels of neophobia, according to the Child Food Neophobia Scale (CFNS), were associated with high consumption of vegetables [142].

### 3.5. Micronutrient Restriction

The picky condition reduced the consumption of folates, Mg, K, Vitamin B_1_, B_2_, B_3_, B_6_, D and E [33,134] and decreased the intake of Fe, carotene, and Zn, which are related to low meat intake, fish, vegetables and fruits [67,133]. Otherwise, when the level of pickiness decreased, the prevalence of Zn deficiency also decreased [60,134].

Among the most notable consequences of FN, there was the need for psychological support, with a prevalence of 37.5% [143]; 60% of parents needed practical support, 47.7% needed emotional support, and 16.2% needed nutritional support [144]. Likewise, high levels of neophobia decreased self-concept at a social, physical and academic level and increased anxiety levels in both children and adolescents [145]. High FN levels were associated with more crying episodes during meals and high food rejection [146]

### 3.6. Results on Food Neophobia Strategies and Treatment

Strategies for FN were discussed to improve pickiness since this disorder altered family and social life [92,143], making difficult relationships between their members and children’s normal development. Different interventions to reduce food neophobia and promote healthy lifestyle habits have been described. There were individual interventions on specific family nuclei and collective interventions at the level of kindergarten or nurseries, as well as at primary and secondary educational centers and on the parents. At the collective level, intervention found improvements in feeding practices, dietary variety, quality, and cognitive children development [146], as well as improvements in the intention to try [147] and the consumption of vegetables after practical intervention in preschool [148,149].

Several strategies were willingness to try tests [141], exposure and reward tests [129], and sensory education programs [150]. Several studies showed that food neophobia and pickiness were decreased by visual exposure to vegetables [151], increasing the desired effect when visual, tactile and sensory exposure were considered [152].

### 3.7. Other Minor Results

The increased risk of picky eating in children was found in mothers who smoked during pregnancy [153]. Meal timing was not associated with fussiness about food [154] and decreased risk of picky eating with moderate physical activity, adequate sleep, and less than 2 h of screen exposure per day [95]. The higher cognitive development, the lower the levels of neophobia [155].

## 4. Discussion

Food neophobia (FN) is the resistance or refusal to eat and/or try new foods, and picky or selective eating is the refusal to eat food, or they eat the same foods over and over [1,9]. Prevalence and incidence of picky eating have been reported as higher than those of FN. The differences in the described prevalence and incidence between FN and pickiness can be understood due to the different severity between FN and pickiness, where the former (FN) has more symptoms than the latter (pickiness), but also that FN could be solved spontaneously at early age of children [1], whereas pickiness could remain despite the age of children and adolescents [17]. Moreover, most of the studies on FN and pickiness are cross-sectional and few long-term; therefore, there have been no opportunities yet to study accurately the prevalence of FN and picky eating.

This review reinforces that food neophobia and picky eating are the primary causes of decreased diet quality in children and adolescents since the possible consequences of food neophobia and picky eating is the alteration in the individual’s weight due to the loss of dietary balance through the lack of several important foods [156]. A consequence is the possibility that individuals with FN could suffer a decrease in weight and BMI due to total caloric restriction or, on the contrary, they would increase in weight and BMI due to poor food choices that would cause very pronounced and persistent FN [157]. The studies found calorie restriction and low weight with impact on adherence to the Mediterranean Diet, which protects against most prevalent chronic diseases [158], thanks to its anti-inflammatory and antioxidant components of foods such as fruits, vegetables and fish, whose consumption decreases disorders such as food neophobia and picky eating [68,112,159,160].

The tools available to reduce or eliminate FN are disparate and do not cover the entire population, which must be stratified according to age and origin so that the data will be comparable. FN should be treated from two perspectives: detailed knowledge of the risk factors and their influence and treatment of the consequences of the loss of dietary variety and nutritional balance. Furthermore, FN should be considered a chronic disease or disorder, which can last more than two years in 40% of cases [18].

Today the risk factors surrounding FN and pickiness are unclear, its long-term consequences nor the main strategies to avoid or minimize it, but there is evidence that childhood eating behaviors often predict eating behaviors in adults. The research carried out so far has concluded that overcoming the biological, familial, environmental and social factors that promote these behaviors will contribute to minimizing their prevalence and long-term consequences [161].

Sex and body weight showed little influence, and age had a moderate influence on food neophobias but is a risk factor in pickiness, mostly in children than in adolescents [50,77,78,79,82,112].

Breastfeeding and its duration have been declared a conclusive risk of FN and pickiness; the longer breastfeeding, the lower the prevalence of FN or pickiness [88,89]. There is no conclusive evidence yet on the effect of the introduction of solid or semi-solid foods into the child’s diet on FN and pickiness [27,32,90].

The literature highlights the need to address environmental and modifiable risk factors to balance the genetic expression of FN and picky eating and eating education, and coercive practices at the family level is the starting point for parents to make correct food decisions regarding their children and adolescents [86]. Parental neophobia should also be considered [77].

Once the teaching process is saved, secondary risk factors (exposure to a variety of foods, environments with greater food insecurity, socioeconomic level, educational level, smoking habit, sleep time, screen time, physical activity or others) would be more easily tackled through collective interventions.

The dietary approach through wide exposure to a variety of foods is used to diminish the aversion to most neophobic foods, such as fruits and vegetables, especially at early ages, with the intention that current social habits regarding the consumption of food with high energy density and scale or no dietary quality, are limited due to both parental education and government strategies that increase the possibility of consuming healthy foods.

Non-modifiable factors such as heritability should, likewise, be able to be counteracted by education and positive and healthy food environments. The strategies must be aimed at the family environment in its entirety, from parental habits (feeding during pregnancy, established lifestyle habits…) to the strategies used from the first day of birth (from the type of breastfeeding, the introduction of solid foods and different textures, to parents’ teaching). At the level of social factors external to the family, actions and strategies addressed to promote healthy lifestyle habits and make difficult access to unhealthy foods and eating practices are encouraged, especially in an age group as vulnerable as children and adolescents.

The need for collective interventions in terms of learning strategies and emotional management of hedonism and aversion to food is unquestionable due to the high prevalence data of these disorders, which emanate from most of the studies reviewed, their persistence in adulthood and their consequences on health. Most of the educational programs and related activities found in the literature addressed the problem of familiarity and exposure to foods in the interest of finding and creating positive eating environments [162]. The globalization of food cultures would open the door to familiarization with non-native foods, and, given that exposure, the effect of rejection of both known and unfamiliar foods would be naturally limited [133]. Familiarity with foods and educational activities are suggested as useful in decreasing food neophobias among children and adolescents [112].

In summary, at the family level, the main interventions to reduce or avoid FN and picky eating should be addressed in three aspects: parental and children–adolescents co-education, training, oral sensory learning in feeding, and avoiding parental coercive practices, especially at mealtime [163]. At the biological level, sex, body weight, and age had moderate effects on FN and pickiness, whereas breastfeeding and its duration were demonstrated to be a factor in decreasing these eating disorders. At the social level, it is highly encouraged to implement, extend and improve sustainable and healthy food environments through political actions, as well as to promote healthy lifestyle habits [163].

### Strengths and Limitations of the Study

The main difficulties encountered in the study have been differences between the validated scales and the specific and isolated methodologies used in many studies. In the paediatric population, difficulties have been found in validating and using the scales depending on whether they were answered directly by the children or were reported by the parents, as a specific method or by the age of the child. All of them can be considered as biases between the reviewed studies.

## 5. Conclusions

Food neophobia and pickiness developed in childhood are conditioned by risk factors related to familiar, biological, social, and environmental characteristics, as well as family education and skills. Therefore, strategies to minimize or avoid these disorders should be aimed at implementing healthy habits at these levels.

## Figures and Tables

**Table 1:** PICOS used.

Patients	Intervention	Comparison	Outcomes	Study Type
Paediatric population (0–18-year-old)	Acceptance or rejection of foods	Food neophobia/picky eating vs. non-neophobic	New tools to measure neophobia; classification and prevalence of food disorders and related risks; effects of food neophobia and picky eating risk factors; consequences linked to food neophobia; macronutrient and food restriction; micronutrient restriction; food neophobia strategies and treatment; other minor results	Cross-sectional, longitudinal, case-control, cohorts, control trials

**Table 2:** Description of the reviewed studies: Authors, study design, participants (n; age), aims, applied methods, and main results.

Authors	Design	n	Age	Aim	Methods	Results
**New tools to measure neophobia**						
Emmett et al., 2018 [32]	Longitudinal	5758–6608	4–15 mo	To assess the early life factors associated with whether a child mey be PE.	Own methodology	Any difficulty during complementary feeding and late introduction of chopped foods were associated with a higher likelihood of PE. A strong predictor was the child being choosy at 15 months, particularly if the mother was worried about this behaviour.
Wetherill et al., 2022 [44]	Cross-Sectional	164	3–6 yr	Validate n of the Farfan-Ramirez WTT (FR-WTT) measurement using FRESH study baseline data.	CFNS	The FR-WTT is a method valid to assess young children’s eating intake of vegetables, as well as behavior in front of them.
Jani et al., 2020 [45]	Cross-Sectional	369	7–12 yr	Development and validation of a parent-reported Picky Eating Questionnaire (PEQ) and child-reported Food Preference Questionnaire (C-FPQ) to investigate environmental and phenotype determinants of picky eating.	PEQ C-FPQ	These tools can be used: to understand parental perceptions of picky eating identify children’s self-reported food preferences.
Johnson et al., 2018 [46]	Multicenter	233	3–5 yr	Development of the Trying New Foods Scale.	TNFS	TNFS validated and adjusted for age and sex.
Damsbo-Svendsen et al., 2017 [47]	Cross-Sectional	235	9–13 yr	Development of novel tools (FNTT) useful for child’s food neophobia measurement.	FNTT	six- and nine-item FNTT has high validity. Internal consistency of the FNTT was higher relative to the FNS.
Steinsbekk et al., 2017 [48]	Cross-Sectional	1035	4–6 yr	To assess the antecedents of pickiness.	CEBQ	Prevalence of PE: 26%. From preschool to school age, pickiness is slightly stable. No association with temperamental surgency and negative affectivity.
Hollar et al., 2013 [49]	Cross-Sectional	1485	8–10 yr	Development and measurement of student attitudes toward new fruits and vegetables: The Development of the Fruit and Vegetable Neophobia Instrument (FVNI).	FNS	The self-administered 18-item questionnaire adapted from the FNS was useful to assess fruit and vegetable neophobia in third–fifth-grade students. Two subscales were described: willingness to try new fruits in different circumstances and the same for vegetables.
Rubio et al., 2008 [50]	Cross-Sectional	166	5–8 yr	To develop and validate a self-report questionnaire on FN in French children.	QENA	Satisfactory internal consistency and good test–retest reliability; a total of 13 items by conducting a Varimax rotation.
Loewen et al., 2000 [51]	Cross-Sectional	335	7–12 yr	To develop and validate of the Food Situations Questionnaire (FSQ) as self-report measurement of FN for children.	FNS FAS	Self-reported questionnaire with 10 items and two sub-scales: willingness to try novel foods in highly stimulating and non-stimulating circumstances. This test predicts willingness to taste novel foods better than parents’ reports of child’s FN.
Pereboom et al., 2023 [53]	Longitudinal	814	3–20 yr	Examine longitudinal associations of picky eating in early childhood with consumption of various foods, and weight status (body mass index, BMI).	CFQ	NP and IMC showed a non-significant association. Increased level of pickiness was observed at 4–5 years, and lower frequency consumption of healthy foods.
de Andrade et al., 2017 [54]	Cross-Sectional	132	15–19 yr	To evaluate the association of taste-related factors (craving for sweets, using food as a reward and pleasure) and FN with nutritional status and food intake among teenagers.	FNS	Craving for sweets was associated with overweight, adiposity, meal skipping, physical inactivity, and intake of sweets. Reward was linked to adiposity, physical inactivity, lack of interest in information about food, and intake of sweets. Pleasure was associated with physical inactivity, lack of interest in information about food, and intake of sweets and soft drinks.
**Classification and prevalence of food disorders and related risks**						
Jones et al., 2010 [52]	Cross-Sectional	7285	7 yr	To examine the sociodemographic, parental and child factors that predict fruit and vegetable consumption	Diet assessed using 3 × 1 R day unweighted food diaries	Consumption of fruit and vegetables appears to be influenced by parental rules about daily consumption and parental consumption and by the child’s choosiness.
Białek-Dratwa et al., 2023 [55]	Cross-Sectional	585	2–7 yr	To assess the prevalence of feeding problems with Montreal Children’s Hospital Feeding Scale.	MCHFS	Groups with the lowest risk feeding problems, risk 0, comprised 445 children (76.06%); group 1, middle difficulties, 59 children (10.08%); group 2, moderate difficulties, 40 children (6.84%); and group 3, most difficulties, 40 children (7.01%).
Diamantis et al., 2022 [56]	Cross-Sectional	2550	5.5–8.5 yr	Assessing persistent PE to predict eating difficulties.	Own methodology	Prevalence: 5% non-persistent PE and 8% persistent PE. There was a decrease in persistent probability with age.
Viljakainen et al., 2019 [57]	Cross-sectional	5700	9–12 yr	To assess the associations of PE and FN with weight status.	FFQ	Prevalence of PE: 34%. Prevalence of FN: 14%. PE was inversely associated with FN.
Grulichova et al., 2022 [58]	Longitudinal	2068	1–15 yr	To evaluate whether preschool children identified as PE showed differences in anthropometric characteristics from their non-PE.	Own methodology	Prevalence of PE: 16.7%. PE had a lower weight (−2.3 kg) and height (−0.8 cm) than non-PE in 15-year-old-adolescents.
Zohar et al., 2020 [59]	Longitudinal	109	3–8 yr	To assess the prevalence of PE in 3–8-year-old-children, as well as to characterize PE children with mothers.	CEBQ	Picky eating persisted in 22.5% of PE children.
Chao et al., 2021 [60]	Cross-Sectional	203	4–7 yr	To assess the relationship between trace element (Fe, Zn, Cu) deficit and PE behavior, developmental and physical activity level.	Based on the Bayley-IV scale	Prevalence of PE: 67.5%. Low level and prevalence of Zn deficiency.
Fernandez et al., 2020 [61]	Cross-Sectional	317	4–9 yr	To assess child picky eating in low-income and associations with participant characteristics, including child BMI z score (BMIz) and maternal feeding-behavior trajectories.	CEBQ CFQ CSFQ	Prevalence of PE: 29% persistently low, 57% persistently medium, and 14% persistently high, which were associated with lower BMIz.
Sandvik et al., 2018 [62]	Cross-Sectional	1272	3–6 yr	To assess the prevalence and characteristics of PE in preschool-aged children with thinness, normal weight, overweight or obesity	CEBQ	Prevalence of PE: severe 30%. Slowness in eating was not as pronounced among PE in the obesity group. Parents of PE were more likely to report their children had too much screen time, complained about physical activity, and expressed negative affect toward food.
Steinsbekk et al., 2017 [63]	Cross-Sectional	752	6 yr	To screen efficiency of the six-item ’Food Fussiness’ (FF) scale from the Children’s Eating Behavior Questionnaire by means of structured psychiatric interviews.	FF-CEBQ	Prevalence of PE: 74.2% no PE, 20.9% moderate PE, and 4.9% severe PE. Higher PE, showed lower BMI. Parental socioeconomic status was unrelated to PE.
Chao et al., 2017 [64]	Cross-Sectional	600	1–10 yr	To assess eating behaviors between PE and non-PE, and finding correlation between parental management of children’s eating disorders with children development.	Own methodology	Prevalence of PE: 62%. High PE was associated to a lack of appropriate caregiver–child interactions, and the presence of inappropriate parental interactions.
Machado et al., 2016 [65]	Cross-Sectional	959	1.5–6 yr	To assess PE prevalence according to the food avoidance or restriction.	CBCL	The prevalence of PE was 25.1%. There were association between picky eating, pregnancy and birth disorders.
Xue et al., 2015 [66]	Cross-Sectional	793	7–12 yr	To assess PE prevalence and associations with school-age children growth.	Own methodology	The prevalence of children picky eating reported by parents was 59.3% in.
Xue et al., 2015 [67]	Cross-Sectional	937	3–7 yr	To find associations between PE and growth and development of pre-schoolers.	FFQ	Prevalence of PE reported by parents was 54%. Last two years PE was associated with low weight for age. PE consumed few cereals, vegetables, and fish, and showed low protein, dietary fibre, iron, and zinc dietary intake.
Rodríguez-Tadeo et al., 2018 [68]	Cross-Sectional	1491	8–18 yr	To assess the impact of FN on adherence to the Mediterranean diet and on the hedonic acceptance of healthy foods made in gastronomic workshops by schoolchildren.	FNS	Prevalence of FN: 13.5%, and 61.1% showed optimal diet quality. Higher the FN, lower the quality of the diet. High adherence to the Mediterranean diet was associated with lower neophobia and better hedonic scores.
Rodríguez-Tadeo et al., 2015 [69]	Cross-Sectional	242	8–12 yr	To identify the impact of FN in food habits and preferences of healthy food in school canteens users.	FNS for spanish population	Prevalence of FN: 16%. Without difference by sex, academic year, time to use service, parental origin and being overweight or underweight.
Kozioł-Kozakowska et al., 2018 [70]	Cross-Sectional	325	2–7 yr	To assess the prevalence of FN in pre-school children and association between FN with eating habits, dietary intake, and anthropometric parameters.	CFNS	Prevalence of FN: 12.3% low FN and 10.8% high FN. High FN showed less likely (*p* < 0.05) to eat eggs, raw or cooked vegetables and legumes. Low FN tended to eat sweets and snacks more frequently.
Galloway et al., 2005 [71]	Cross-Sectional	173	8–9 yr	To evaluate whether mothers’ fruit and vegetable intake and their pressure on the diet of 7-year-old daughters predicted PE and those intake at 9 years of age, analyzing diet and body weight according to whether they were PE or non-PE.	CFQ FFQ	Mothers who ate more fruits and vegetables had daughters who were less picky and ate more fruits and vegetables, and put less pressure on their daughters to eat. PE ate less fruits, vegetables, fats, and sweets.
Sdravou et al., 2021 [72]	Cross-Sectional	742	2–7 yr	To determine the prevalence of feeding disorders in young Greek children.	Child feeding behavior, and Behavioral Pediatrics Feeding Assessment Scale.	Most of the analyzed pediatric population showed high frequency of favorable and specific child feeding behaviors that could be improved.
Cardona et al., 2016 [73]	Cross-Sectional	3748	1.5, 3, and 6 yr	To distinguish between children with PE vs persistent PE behavior in adverse outcomes.	Child’s behaviors were assessed with the Teacher’s Report Form.	Persistent PE may be symptom/sign of general developmental problems, but does not predict behavioral problems. Adverse mental health was not associated with remitting PE.
**Effects of FN and picky eating risk factors**						
Trofholz et al., 2017 [6]	Cross-Sectional	88	2–18	To describe parent’s and children’s eating practices and behaviors.	Own methodology	Children were frequently described as being PE. Parents defined picky eating in a variety of ways. PE impacted on the family meal. Parents responded to PE in a variety of ways.
Hafstad et al., 2013 [22]	Longitudinal	476	1.5–4.5 yr	To describe the development and examine predictors of PE from 1.5 to 4.5 years of age.	Own methodology	PE increased significantly from 1.5 to 4.5 years. Lower maternal age, higher levels of child emotionality, and maternal negative affectivity at the child’s age 1.5 predicted an increase in PE from 1.5 years to 2.5 and 4.5 years.
Galloway et al., 2003 [26]	Cross-Sectional	192	7 yr	To assess if food neophobia and pickiness contribute to low vegetable intake in school-aged girls, looking for FN and PE predictors.	CFQ	Girls with both FN and pickiness consumed fewer vegetables than girls with neither FN nor pickiness. The picky girls had mothers with low vegetable consumption and that perceived their family with scarce time to eat healthy foods.
Shim et al., 2011 [27]	Longitudinal	129	3 yr	To assess the effect of preschoool child feeding practices on the PE behavior development.	CEBI	Children who received complementary foods before 6 months of age showed 2.5 higher odds of developing FN and scarce food variety.
Smith et al., 2017 [74]	Cross-Sectional	1291	16 mo	To determine the contribution of genetic and environmental influences on PE and FN. To determine how much they share genetic and environmental influences.	CEBQ	Food fussiness and FN were strongly correlated. Proportions of variation in PE were equally explained by genetic and shared environmental influences.
Cooke et al., 2007 [75]	Cross-Sectional	5390	8–11 yr	To determiine the effect of environmental and genetic factors on child FN.	CFNS	NF is highly heritable. No influence of shared environmental factors.
Faith et al., 2013 [76]	Cross-Sectional	132	4–7 yr	To examine whether FN is associated with parent–child feeding relations or child body fat.	CFNS	Heritability of PE= 72%.
Rahill et al., 2018 [77]		594	5–12 yr	To define the determining factors of food fussiness, if the child’s age determines these factors, and to identify if parental neophobia determines independently the food fussiness.		The age of the child determines the how much factors influence food fussiness and that parental neophobia is an independent determinant of it.
Yuan et al., 2016 [78]	Longitudinal	1142	0–5 yr	To assess the food-related characteristics in the first years of life associated with the child’s taste for different foods at 5 years of age.	Own methodology	High FN at 4 years of age was related to low child’s taste for all food groups. Maternal feeding practices at 2 years of age were negatively associated with practices that allowed the child to control their own food intake, and directly associated with restricting the child’s food intake for weight reasons.
Venkatesh et al., 2022 [79]	Cross-Sectional	95	3–10 yr	To determine the factors of picky eating behavior.	CEBQ	The pickiest children are 3 to 4 years old.
Caldwell et al., 2023 [80]	Cohort	80	6–8 mo	Assessment of Child Pickiness, Parent Anxiety, and Family Meal Structure	MCHFS	Significant association were found between infant feeding problems and child age, parental anxiety, and resistance to trying foods.
Brown et al., 2018 [81]	Cross-Sectional	506	3–4 yr	To determine PE predictors and test the association between PE with dietary quality, micronutrient intake, and children’s body mass index z-score (BMIz).	CEBQ	High PE was associated with male sex, older child age, and more difficult temperament. No association with race/ethnicity, maternal body mass index, maternal depressive symptoms, household food insecurity, or single parent home. Negative association with total Healthy Eating Index-2010 score and servings of whole fruit, total vegetables, greens and beans, and total protein foods.
Roßbach et al., 2016 [82]	Cross-Sectional	166	10–18 yr	To asess FN potential determinants and their association with dietary habits of DONALD study participants.	FNS	Children’s FN was associated with parental FN, and negatively associated with protein intake. The overall low level of FN amy be attributed to the FN low level in adolescents.
Johnson et al., 2015 [83]	Cross-Sectional	249	4 yr	To assess associations between FN, sensory sensitivity, and dietary intake in developing preschoolers.	CFNS	Low scores for children’s oral sensory characteristics were related to high FN. There were no differences for FN by parental education and income, and child sex.
Guzek et al., 2017 [84]	Cross-Sectional	163	10–12 yr	To analyze the association between the FN level and the intake of fruits and vegetables.	FNS	High FN was associated to low intake of vegetables, in both sexes. In girls, differences were observed in vegetable intake according to the NF category.
Kutbi, 2021 [85]	Cross-Sectional	424	6–12 yr	To assess sociodemographic determinants of children PE, and the associations between PE and dietary intake.	FFQ	Greater risk of being PE if the mother was obese. High levels of picky eating were associated to lower consumption of fruits, vegetables and proteins. High levels of picky eating showed increased consumption of trans fatty acids.
Taquet et al., 2024 [86]	Cross-Sectional	157	4–6 yr	To determine the risk factors for neophobia in children related to the parenting practice and personality of the parents.	CFNS	Higher levels of a coercive parenting style were associated with higher levels of both food fussiness and food neophobia.
Chen et al., 2024 [87]	Cross-Sectional	408	3–6 yr	Effect of parenting strategies on children’s health and selective eating behaviors.	CEBQ	Direct effect of parenting strategies and selective eating behaviors.
Vaarno et al., 2015 [88]	Cross-Sectional	1797	4–13 mo	To assess the association of parental feeding behaviour and dietary patterns with eating practices of infants and young children.	FNS	Neophobic mothers breastfed exclusively or shorter times than average mothers.
Olmer Specht et al., 2018 [89]	Randomized controller trial	236	2–6 yr	To determine whether length of exclusive breastfeeding was associated with pickiness, and intake of vegetables, fruit, starchy foods, and sugar sweetened beverages.	Own methodology	Exclusively breastfed until 4–5 months of age showed lower probability of PE than exclusively breastfed for 0–1 months.
Bell et al., 2018 [90]	Cross-Sectional	206	1–5 yr	To investigate associations between whole dietary patterns in young children, and breastfeeding duration, age of solid introduction and FN.	CFNS	Shorter breastfeeding duration and poorer child food neophobia scores were associated with higher dietary risk scores. Age of introduction to solids showed no association with dietary risk.
Wolstenholme et al., 2022 [91]	Cross-Sectional	16	7–10 yr	To explore how children perceived, experienced and managed fussy eating behaviours, and how children experienced feeding dynamics according to these behaviours.	CEBQ	Children described tension between food internal experiences and external expectations. Children were aware of parental strategies and goals and develop their own anti-aversion strategies.
Chilman et al., 2023 [92]	Cross-Sectional	10	2–6 yr	To explored parents’ experiences of parenting an extremely picky eater.	CEBQ	Picky eating impacts families and mealtimes. Parents have attempted multiple strategies to manage picky eating. Emotions associated with parenting an extremely picky eater.
Lumeng et al., 2018 [93]	Cross-Sectional	222	21–33 mo	To examine the cross-lagged associations between mother-reported pressuring feeding, mother-reported child PE and measured weight-for-length z-score.	CEBQ IFSQ	Associations between pressuring feeding and PE Prospective associations between pressuring feeding and future WLZ.
Moding et al., 2016 [94]	Longitudinal	82	18 mo–4.5 yr	To investigate the association between FN and the temperamental processes of parental approach/withdrawal on children.	CFNS	Child temperament was associated with FN. The FN gets stronger by a maternal pressure of eating behavior.
Yalcin et al., 2022 [95]	Cross-Sectional	913	6–13 yr	To assess the relationships of children’s picky eating with dietary habits, physical activity, sleep, and screen time.	Own methodology	The risk of picky eater is increased if the children’s parents are fussy. The risk decreases with ≥1 h/day physical activity, ≥9 h /day sleep time, and ≥2 h/day screen time.
Norton et al., 2016 [96]	Case-Control	24	1–2.5 yr	Association between primary caregivers and FN and how food preferences develop in young children.	Own methodology	Young children’s caregivers are unaware of FN, and its association with the development of food preferences.
Coulthard et al., 2016 [97]	Randomized controlled trial	83	18 mo–4 yr	To test and assess the intervention to evaluate if participation in tactile sensory tasks improves fruit acceptance.	CFNS	Children in both the combined sensory play and sensory play without food enjoyed more eating fruits than children without sensory play condition (control). No-food sensory play children enjoyed more eating fruits than the fruit-exposure children.
Moding et al., 2016 [98]	Longitudinal	82	6 mo–4.5 yr	To assess if novel food rejection in infancy predicted child behavioral and parent-reported FN.	CFNS	Children with low rejection at 6 months showed high father-reported FN when mothers showed high levels of FN. Infants who exhibited low levels of rejection at 6 months showed higher levels of parent-rated FN when their mothers also showed high compared to low levels of FN.
Howard et al., 2012 [99]	Randomized Controled Trial	277	24 mo	To identify factors that influence children’s liking for fruits, vegetables and non-core foods.	CFNS	Maternal preferences corresponded with child preferences. FN was associated with liking fewer vegetables and fruits, and trying fewer vegetables. Number of repeated exposures to new food was not significantly associated with food liking at this age.
Tan et al., 2012 [100]	Cross-Sectional	85	3–12 yr	To examine the associations between children’s and mother’s FN and parental feeding practices	FNS CFNS	Mothers with FN children and FN mothers reported to do not make healthy foods available for children. Mothers with high FN also used more restriction for weight.
Wardel et al. [101]	Cross-Sectional	564	2–6 yr	To assess the negative association of fruit and vegetable consumption in girls and boys with parental monitoring, determining sex differences, and testing hypothesis to explain this association.	PCI CFNS	Children’s fruit and vegetable consumption was correlated with parental control with no sex differences. Child’s food neophobia and parental fruit and vegetable consumption were predictors of child’s fruit and vegetable consumption.
Poelman et al., 2019 [102]	Cross-Sectional	299	8–12 yr	To determine the effectiveness of Vegetable Education to Increase Children’s Acceptance and Liking, a sensory education program for schoolchildren.	Own methodology	No effect on FN: Ability to verbalize sensations, high knowledge on vegetables and senses, and acceptance to try vegetables.
Cassells et al., 2014 [103]	Randomized Controlled Trial	244	4 mo	To examine the association of maternal beliefs about infant feeding and the expression of FN and its relationship with the control of feeding practices.	CFQ CFNS	Higher maternal Concern about infant under-eating and becoming underweight at 4 months was associated with higher child FN at 2 years. High FN showed lower awareness of infant hunger and satiety.
Cooke et al., 2003 [104]	Cross-Sectional	564	2–6 yr	To examine the contribution to fruit and vegetable eating in children of potential predictive variables within the domains of demographics, parental feeding practices and personality traits.	CFNS	Demographic variables were associated with child’s vegetable consumption, mother’s education and child’s age and gender. Two characteristics of children themselves (FN and food enjoyment) were highly associated to fruit and vegetables consumption.
Russell et al., 2013 [105]	Cross-Sectional	371	2–5 yr	To investigate the relationship between children’s food preferences and FN, personality, food attributes (texture and appearance) and socialization experience.	CFNS	Results provided preliminary evidence of differences in parents’ attributions and self-efficacy beliefs in the feeding domain. Highlighting the need for greater understanding of the ways in which parents’ beliefs affect children’s food preferences.
Jordan et al., 2020 [106]	Longitudinal	199	5–7 yr	To test the correlation of counts of maternal prompting types with child vegetable intake, and picky eating. To assess the interaction of stimulus and PE on vegetable consumption.	CFSQ PFQ CEBQ FNS	Positive influence of the type of education and the consumption of vegetables. Higher use of Autonomy Promotion-Praise was directly correlated with amount of green beans eaten.
Cole et al., 2018 [107]	Cross-Sectional	497	3–5 yr	To identify the association between factors of the home feeding environment with PE behavior.	Own methodology	High PE was related to child control over feeding and watching television during mealtime. Low PE was related to favourable family meal environment and mealtime habits.
Jacobi et al., 2008 [108]	Cross-Sectional	426	8–12 yr	To examine the prevalence of PE and the relationship between PE.	SFQ	PE were reported to avoid foods in general more often than non-PE. PE displayed more altered behaviors comprising both internal and external behaviors.
Jarman et al., 2015 [109]	Longitudinal	288	2–5 yr	To examine the role of FN in predicting changes in control practices, and to look at associations between these factors and children’s quality of diet.	CFNS	Mothers’ covert control of their children’s diet improved diet quality.
Tharner et al., 2014 [110]	Cohort	4914	4 yr	To identify an eating behavior profile reflecting fussy/PE in children and to describe characteristics of fussy eaters.	CEBQ	A total of 5.6% “fussy” eating behavior profile. Fussy eaters were more often from families with low household income than non-fussy eaters. Picky eaters had higher probabililty to be underweight at age 4 yr.
Harris et al., 2019 [111]	Cross-Sectional	260	2–5 yr	To determine the influence of food insecurity on food exposure practices.	CEBQ	Mothers reporting food insecurity (11%) had less fruit availability at home than those reporting food security.
del Campo et al. 2023 [112]	Cross-Sectional	600	11–18 yr	to assess the preferences for tastes and foods and food neophobias among Spanish adolescents and to compare the differences between boys and girls.	Food Taste Assessment, and FNS	Spanish adolescents showed preference for sweet, salty, and umami tastes, as well as food neophobia towards foods that they do not regularly consume, mainly those with a bitter taste. Gender and body weight showed little influence and age-moderate influence on food neophobias.
Monnery-Patris et al., 2015 [113]	Cross-Sectional	123	20–22 mo	To assess the relationships between smell and taste differential reactivity and to determine the relationships between smell (or taste) differential reactivity and FN.	Own metodology	Smell and taste differential reactivities were not correlated. FN scores were modestly but significantly positively correlated with smell differential reactivity but not with taste differential reactivity. The smell reactivity and FN were correlated only among boys.
Coulthard et al., 2009 [114]	Cross-Sectional	73	2–5 yr	To ascertain the relative contribution of FN and taste sensitivity to the amount of fruit and vegetables consumed in a typical day.	CFNS CFQ	Parental and child fruits and vegetables consumption in the sample were positively associated. Children sensitive to taste/olfactory stimuli consumed less fruit and vegetables, despite fruits and vegetables consumption of their mothers.
Coulthard et al., 2015 [115]	Cross-Sectional	70	2–5 yr	To assess the association between children’s enjoyment of tactile play and FN, and fruits and vegetables consumption.	FNS	Significant association between game enjoyment and lower FN. Significant association of childhood fruit and vegetable consumption with parental fruit and vegetable consumption. No association of FN with tactile enjoyment.
Smith et al., 2005 [116]	Cross-Sectional	62	3–10 yr	To explore whether tactile defensive children have PE habits.	Own methodology	Tactile defensive children had scarce/poor appetite, neither consumed unfamiliar foods, nor at other people’s houses, refused some foods due to their smell and temperature, and showed problems consuming vegetables.
Maratos et al., 2015 [117]	Clinical Trial	70	8–11 yr	To investigate attentional biases to familiar and unfamiliar fruit and vegetables with levels of FN.	FNS	Visual aspects of food stimuli (e.g., familiarity) play an important role in childhood FN.
Monneuse et al., 2008 [118]	Cross-Sectional	39	10–18 yr	The relationship between taste acuity and FN, food familiarity and liking.	FNS FFLQ	FN limited reductions were related with taste acuity. High acceptability of healthy foods, mainly fruits and vegetables was reported in less sensitive subjects.
**Consequences linked to FN**						
van der Horst et al., 2016 [110]	Cross-Sectional	2371	1–4 yr	To determine associations between, toddlers’ PE, eating characteristics, and food consumption.	Own methodology	Toddlers showed high FN to certain textures. Eggs, burritos/tacos/ enchiladas /nachos, and sandwiches consumption was lower in PE than in non-PE. PE consumed fewer vegetables and raw vegetables than non-PE.
Berger et al., 2016 [119]	Cross-Sectional	181	5–15 yr	To assess whether childhood PE was associated to parental pressure, nutrition, and growth over 10 years.	CFQ	Persistent PE (18%) had lower BMI. Children who persistently picked at eating were less likely to be overweight as teenagers. Children who persistently picked at eating also received greater pressure to eat.
Sandvik et al., 2019 [120]	Randomized controlled trial	130	4–6 yr	Examines the role of picky eating.	CEBQ LBC	Association of initial selective feeding, with lower BMI and lower consumption of vegetables. Children with a higher degree of picky eating at baseline displayed a lower degree of weight loss.
Antoniou et al., 2016 [121]	Longitudinal	1024	5–9 yr	To determine the association between body weight status and PE, as well as the moderating role of parental dietary practices.	Own methodology	At 5 years old, the pickiest children were slightly shorter, less overweight, with equal energy intake according to body weight. The association between child weight status and picky eating was not altered by parental practices.
Ekstein et al., 2010 [122]	Cross-Sectional	170	0–3 yr	To assess the relationship of PE with underweight status in children.	Own methodology	Prevalence of PE: 206% PE and 6.6% underweight.
Taylor et al., 2019 [123]	Longitudinal	1856	7–17 yr	To determine whether PE children showed different body weight, height, and composition vs. non-PE.	Own methodology	Effect of being PE on height, weight, BMI, and lean mass, but no effect on body fat percentage and fat mass index has been described. Being a PE predict being thin at certain age points.
Brown et al., 2020 [124]	Cross-Sectional	286	2–8 yr	PE assessment based on parental perception of their child’s weight status, parental pressure-to-eat, and BMI z-score.	CEBQ CFQ CFNS PEQ	No factors were found to related with weight perception. Parents who were more concerned about whether their children were eating enough were more likely to pressure them to eat, and children had lower BMIz.
Knaapila et al., 2014 [125]	Longitudinal	2191	13 mo	To determine if FN is associated with lower overall dietary quality and higher BMI.	FNS	Higher FN is associated with lower consumption vegetables. FN can alter adaptation to dietary recommendations, predisposing to overweight.
Finistrella et al., 2012 [126]	Cross-Sectional	140	2–6 yr	To investigate cross-sectional associations of FN and PE in preschoolers and in their mothers with regard also to food consumption, proposal of new foods, feeding, and weaning modes.	CFNS FNS	Significant relationship between FN and the pickiness of mothers and children. Overweight and obese children were significantly more neophobic and pickier than normal-weight children.
Li et al., 2017 [127]	Cross-Sectional	1414	6–35 mo	To assess the PE prevalence in Chinese children, finding how parents’ perceptions of PE are related with children’s food consumption and body composition.	Own methodology	PE was found in 36% of 24–35 month-old children compared to 12% in those aged 6–11-month-old. No differences were found in nutrient dietary intake between PE and non-PE.
Jacobi et al., 2003 [128]	Cross-Sectional	135	0–5.5 yr	Validation of PE parent-reported concept through laboratory-based objective measures, and identifying precursors and concomitants of PE both child and parents.	SFQ CBQ	PE children ate less foods, mainly avoided vegetables. The caloric intake of 3.5 and 5.5-year-old picky eating girls was reduced, while all other children increased it.
Perry et al., 2015 [129]	Cross-Sectional	330	24 mo	Relationship between FN in 24-month-old children and body weight, consumption of several foods, and variety of fruit and vegetables.	CFNS	The lowest variety of fruits and vegetables were found at 24 months in more neophobic children. FN was associated with poorer dietary quality.
Costa et al., 2020 [130]	Cross-Sectional	150	3–13 yr	To assess FN and eating behavior in children and adolescents from different age groups, and age and sex specific body mass index.	CEBQ CFNS	Adolescent FN was associated with low consumption and preference for specific foods, with no impact on a healthy dietary pattern.
van der Horst, 2012 [131]	Cross-Sectional	305	6–12 yr	To examine whether increasing eating enjoyment and cooking enjoyment might give opportunities to decrease PE.	CEBQ CFQ	Strong inverse association between eating enjoyment and PE. Significant direct effects were found between cooking enjoyment and PE and restriction and PE
**Macronutrients and food restriction**						
Falciglia et al., 2000 [33]	Cross-Sectional	70	8–10 yr	To determine whether children with food neophobia have more restrictive diets than children without FN.	FNS	Fewer FN children met 2/3 of the recommended vitamin E. FN children showed higher intake of saturated fat and lower food variety than non-FN children.
Li et al., 2022 [132]	Cross-Sectional	879	1–3 yr	To assess the relationship between picky eating, frequency of sugared foods and sugar-sweetened beverages consumption, and daily screen time.	FFQ	Children with PE and daily screen time were associated with frequency of added sugar consumption. Children with PE consumed sugary foods and drinks more frequently.
Taylor et al., 2016 [133]	Cross-Sectional	815	2–7.5 yr	To quantify intakes of nutrient and food group in PE and non-PE children, comparying with UK reference nutrient intakes.	FFQ	PE aged 3 years had lower mean carotene, iron, and zinc intakes than non-picky eaters. No significant differences in energy intakes. Nutrient differences were explained by lower intakes of meat, fish, vegetables, and fruits in PE than in non-PE. Older PE showed the highest intakes of sugary foods and drinks.
Gan et al. [134]	Cross-Sectional	321	7–10 yr	To determine the association between children’s PE and nutrient intake from home-prepared school lunches.	CFNS FNS PE FFQ	PE affected the food intake of children who prepared linch at home. Low nutritional quality was obseved in many home-packed lunches.
Dubois et al., 2007 [135]	Longitudinal	2103	2.5–4.5 yr	To determine associations between eating behaviours (PE, irregular eating and overeating), and dietary adequacy according to nutritional recommendations and body weight in preschool.	Own methodology	PE children consumed less total fat, energy, and protein. PE were more likely to consume less fruit and vegetables, and meat and alternatives than dietary recommendations.
Guzek et al., 2018 [136]	Case-Control	1014	12–13 yr	To analyze the association between FN level and the intake of fruits and vegetables.	FNS	High FN level is associated with a lower fruit and vegetable intake. At the same level of FN with an active lifestyle and urban areas were observed high fruit consumption, and sedentary behavior; and those from suburban áreas showed low fruit consumption.
De Wild et al., 2018 [137]	Cross-Sectional	750	2–6 yr	To assess whether breastfeeding duration predicts vegetable intake.	CEBQ CFNS	High FN was associated to children’s vegetable intake. Low FN was associated to vegetable liking and intake.
Mustonen et al., 2012 [138]	Cross-Sectional	208	8–11 yr	To assess if food familiarity and affective responses to food are predicted by FN traits and parental education in school-aged children.	FNS	Low FN children were familiar with a higher number of foods. High FN was related with low pleasantness of most foods, including cheese, fruit and vegetables, fish, starch and cereals, and ethnic and exotic foods.
Russell et al., 2008 [139]	Cross-Sectional	371	2–5 yr	To investigate the relationships between food preferences, FN, and children’s characteristics.	CFNS	FN was related with low preferences for food groups, vegetables, and associated with liking fewer food types, low number of untried food types, low varied range of food preferences, and low healthful food preferences overall.
Kähkönen et al., 2018 [140]	Cross-Sectional	130	3–5 yr	To assess the association between mother’s education level and sensory-based food education implemented in early childhood education and care (ECEC) centres and children’s willingness to choose and eat vegetables and fruit with FN.	FNS	Sensory-based food education was associated with children’s willingness to choose and eat vegetables and fruit. High FN, reduced the children’s willingness to choose vegetables and fruit.
Tuorila et al., 2010 [141]	Cross-Sectional	72	8–11 yr	To examine the reluctance vs. willingness to try a newfood as a predictor of pleasantness ratings of this food.	FNS	Children who like to try a food, they rated it as pleasant. Children who did not like to try a food, but tasted it, rated it as non-pleasant and similar to those they did not like to try, without previous tasting. FN evaluated by parents was associated to willingness to try.
Tsuji et al., 2011 [142]	Cross-Sectional	323	4–6 yr	To investigate whether intake of vegetables, fruits and soy foods is associated with sensitivity to bitterness and reluctance to eat new foods (FN) in Japanese preschool children.	CFNS	A high intake of vegetables was significantly associated with a low CFNS score in boys. High intake of soy foods was significantly associated with a low NF score in PROP tasters but not in non-tasters
**Micronutrient restriction**						
Fernández et al., 2022 [143]	Cases and controls	133	0–12 yr	To determine the impact of children eating disorders on the quality of life, stress and psychological health of families.	PSI-SF and GHQ-28	A total of 47.9% did not acomplished the paediatrician’s recommendations, 54.2% declared limitations on social life, 25% showed altered relationships, 47.9% feeling themselves as externally observed, and 37.5% having sought or considered seeking psychological support.
Fraser et al., 2021 [144]	Cross-Sectional	130	1–3 yr	To assess parental feeding concerns and support their assistance to improve parental responsive feeding practices.	Own methodology	A total of 60% of parents sought practical support, 47.7% of parents sought emotional support, and 16.2% of parents sought food support.
Maíz et al., 2018 [145]	Cross-Sectional	831	8–16 yr	To determine whether children and adolescents with FN differed in trait anxiety and dimensions of self-concept.	CFNS (Spanish version)	High level of FN is asociated with higher levels of trait anxiety, and lower social, physical, and academic self-concept.
Blomkvist et al., 2018 [146]	Randomized controller trial	210	1 yr	To develop, measure and compare the effect of two different interventions to reduce FN and promote healthy diets.	CFNS	The intervention in kindergartens and parents would provide improvements in feeding practices, improvements in the dietary variety improvement in quality, cognitive development and weight reduction.
**FN strategies and treatment**						
Allirot et al., 2016 [147]	Cross-Sectional	137	7–11 yr	To evaluate the children involvement in cooking on their willingness trying new foods, and improvement of food consumption, taste and hunger.	Own methodology	A high involvement in cooking improves the dietary quality, taste of unfamiliar foods in children.
De Wild et al., 2017 [148]	Randomized Controlled Trial	103	2–4 yr	To compare vegetable preparation practices on increasing vegetable preference and intake.	CFNS	Increase in vegetable consumption by 70% from before to after the intervention. The vegetable consumption depends on the children FN status before an intervention.
Laureati et al., 2014 [149]	Clinical Trial	560	6–9 yr	To assess the effectiveness of the ’Food Dudes’ school-based intervention consisting of rewards and food exposure on FN and the liking of fruits and vegetables.	FNS	The intervention was effective in reducing food neophobia. Persistent effect was observed 6 months.
Reverdy et al., 2008 [150]	Randomized Controlled Trial	180	8–10 yr	To determine the effect of a French sensory education program on the food habits of school children.	FNS WTNF	At the end of the education period, FN decreased, and willingness to try new foods increased.
Rioux et al., 2018 [151]	Randomized Controlled Trial	70	3–6 yr	To assess the effectiveness of visual exposure to vegetables in young children on FN and pickiness decrease.	Own methodology	Visual exposure led to high consumption of exposed and unexposed vegetables.
Coulthard et al., 2017 [152]	Cross-Sectional	62	3–4 yr	To determine if engaging in a sensory play activity with real fruits and vegetables can estimulate tasting in preschool children.	CFNS	Sensory play activities with fruits and vegetables encouraged the taste of fruits and vegetables much better than other activities in preschool children.
**Minor results**						
Bourne et al., 2023 [153]	Longitudinal		2–10 yr	To investigate the prevalence of childhood picky eating (PE) and to identify risk factors associated with different PE trajectories.		High risk of picky eating from mothers who smoke during pregnancy and children with feeding problems between 9 and 12 months.
Searle et al., 2020 [154]	Cross-Sectional	205	2–5 yr	To assess relationships of mothers’ and fathers’ structure-related feeding practices with child temperament, as well as if these associations were child fussy eating-mediated.	FPSQ-28 STSC CEBQ	Low perceived as PE if easier temperament. Structured meal timing was not significantly associated with food fussiness.
Pickard et al., 2023 [155]	Cross-Sectional	237	3–7 yr	To investigate at what age children have sufficient script and thematic knowledge in the food domain and its relationship with neophobia.	Own methodology	The greater the cognitive development, the lower the FN.

**Abbreviations:** CBCL: Child Behavior Checklist; CEBI: Children’s Eating Behavior Inventory; CEBQ: Child Eating Behavior Questionnaire; FPQ: Child-reported Food Preference Questionnaire; CFQ: Child Feeding Questionnaire; CFRS: Child food rejection scale; CFSQ: Caregiver’s Feeding Styles Questionnaire; FAS: Food Attitudes Scale; FFLQ: Food Familiarity and Liking Questionnaire; FFQ: Food frequency questionnaire; FN: Food Neophobia; FNS: Food Neophobia Scale; FNTT: Food Neophobia Test Tool; FR-WTT: Farfan-Ramírez Willingness To Try; FSQ: Food Situations Questionnaire; FVNI: Fruit and vegetable neophobia instrument; FVNI: Fruit and Vegetable Neophobia Instrument; GHQ-28: Goldberg’s Health Questionnaire; IFSQ: Infant Feeding Styles Questionnaire; LBC: Lifestyle Behavior Checklist; MCHFS: Montreal Children’s Hospital Feeding Scale; Mo: months of age; PAQ: Parental Authority Questionnaire; PCI: Parental Control Index; PE: Picky Eater; PEQ: Picky Eating Questionnaire; PFQ: Preschooler Feeding Questionnaire; PFQ: Preschooler Feeding Questionnaire; PSI-SF: Parent Stress Index Short Form; QENA: Questionnaire on Food Neophobia among French-speaking children; SFQ: Stanford Feeding Questionnaire; TNFS: Trying New Foods Scale; WTNF: Willingness to Taste Novel Food; YR: years of age.

## Data Availability

There are restrictions on the availability of the data of this trial due to the signed consent agreements around data sharing, which only allow access to external researchers for studies following the project’s purposes. Requestors wishing to access the trial data used in this study can make a request by emailing pep.tur@uib.es.

## Abbreviations

ARFID: Avoidant Restrictive Food Intake Disorder; BMI: body mass index; CBCL: Child Behavior Checklist; CEBI: Children’s Eating Behavior Inventory; CEBQ: Child Eating Behavior Questionnaire; CFNS: Child Food Neophobia Scale; CFPQ: Child-reported Food Preference Questionnaire; CFQ: Child Feeding Questionnaire; CFRS: Child food rejection scale; CFSQ: Caregiver’s Feeding Styles Questionnaire; FAS: Food Attitudes Scale; FFLQ: Food Familiarity and Liking Questionnaire; FFQ: Food frequency questionnaire; FN: Food Neophobia; FNS: Food Neophobia Scale; FNTT: Food Neophobia Test Tool; FR-WTT: Farfan-Ramírez Willingness To Try; FSQ: Food Situations Questionnaire; FVNI: Fruit and vegetable neophobia instrument; FVNI: Fruit and Vegetable Neophobia Instrument; GHQ-28: Goldberg’s General Health Questionnaire; IFSQ: Infant Feeding Styles Questionnaire; LBC: Lifestyle Behavior Checklist; MCHFS: Montreal Children’s Hospital Feeding Scale; Mo: months of age; PAQ: Parental Authority Questionnaire; PCI: Parental Control Index; PE: Picky Eating; PEQ: Picky Eating Questionnaire; PFQ: Preschooler Feeding Questionnaire; PFQ: Preschooler Feeding Questionnaire; PSI-SF: Parent Stress Index Short Form; QENA: Questionnaire on Food Neophobia among French-speaking children; SFQ: Stanford Feeding Questionnaire; TNFS: Trying New Foods Scale; WTNF: Willingness to Taste Novel Food; YR: years of age.

## References

[1] Białek-Dratwa A., Szczepánska E., Szymánska D., Grajek M., Krupa-Kotara K., Kowalski O. (2022). Neophobia—A Natural Developmental Stage or Feeding Difficulties for Children?. Nutrients.

[2] Boquin M., Smith-Simpson S., Donovan S.M., Lee S.Y. (2014). Mealtime Behaviors and Food Consumption of Perceived Picky and Nonpicky Eaters through Home Use Test. J. Food Sci..

[3] Bravo P., Hodgson M.I. (2011). Trastornos alimentarios del lactante y preescolar. Rev. Chil. Pediatr..

[4] Guzek D., Nguyen D., Głabska D. (2021). Food Neophobia and Consumer Choices within Vietnamese Menu in a Polish Cohort Study. Int. J. Environ. Res. Public Health.

[5] Wolstenholme H., Heary C., Kelly C. (2019). Fussy eating behaviours: Response patterns in families of school-aged children. Appetite.

[6] Trofholz A.C., Schulte A.K., Berge J.M. (2017). How parents describe picky eating and its impact on family meals: A qualitative analysis. Appetite.

[7] Boquin M., Moskowitz H.R., Donovan S.M., Lee S.Y. (2014). Defining perceptions of picky eating obtained through focus groups and conjoint analysis. J. Sens. Stud..

[8] Moore S.N., Tapper K. (2010). Murphy S Feeding goals sought by mothers of 3-5-year-old children. Brit. J. Health Psychol..

[9] Predieri S., Sinesio F., Monteleone E., Spinelli S., Cianciabella M., Daniele G.M., Dinnella C., Gasperi F., Endrizzi I., Torri L. (2020). Gender, age, geographical area, food neophobia and their relationships with the adherence to the mediterranean diet: New insights from a large population cross-sectional study. Nutrients.

[10] Drewnowski A. (1997). Taste preferences and food intake. Annu. Rev. Nutr..

[11] Nicklaus S., Issanchou S. (2007). Understanding Consumers of Food Products. Children and Food Choice.

[12] Werthmann J., Jansen A., Havermans R., Nederkoorn C., Kremers S., Roefs A. (2015). Bits and pieces. Food texture influences food acceptance in young children. Appetite.

[13] De Oliveira Torresa T., Gomes D.R., Mattosa M.P. (2021). Factors associated with food neophobia in children: Systematic review. Rev. Paul. Pediatr..

[14] Patel M.D., Donovan S.M., Lee S.-Y. (2020). Considering Nature and Nurture in the Etiology and Prevention of Picky Eating: A Narrative Review. Nutrients.

[15] Kerzner B., Milano K., MacLean W.C., Berall G., Stuart S., Chatoor I. (2015). A Practical Approach to Classifying and Managing Feeding Difficulties. Pediatrics.

[16] Chatoor I. Eating Disorders in Infancy and Early Childhood. The Oxford Handbook of Child and Adolescent Eating Disorders: Developmental Perspectives. https://www.oxfordhandbooks.com/view/10.1093/oxfordhb/9780199744459.001.0001/oxfordhb-9780199744459-e-012.

[17] Taylor C.M., Wernimont S.M., Northstone K., Emmett P.M. (2015). Picky/fussy eating in children: Review of definitions, assessment, prevalence and dietary intakes. Appetite.

[18] Mascola A.J., Bryson S.W., Agras W.S. (2010). Picky eating during childhood: A longitudinal study to age 11 years. Eat. Behav..

[19] van der Horst K. (2012). Overcoming picky eating. Eating enjoyment as a central aspect of children’s eating behaviors. Appetite.

[20] van der Horst K., Eldridge A., Deming D., Reidy K. (2014). Caregivers’ perceptions about picky eating: Associations with texture acceptance and food intake. FASEB J..

[21] Dovey T.M., Staples P.A., Gibson E.L., Halford J.C. (2008). Food neophobia and ‘picky/fussy’ eating in children: A review. Appetite.

[22] Hafstad G.S., Abebe D.S., Torgersen L., von Soest T. (2013). Picky eating in preschool children: The predictive role of the child’s temperament and mother’s negative affectivity. Eat. Behav..

[23] Jani Mehta R., Mallan K.M., Mihrshahi S., Mandalika S., Daniels L.A. (2014). An exploratory study of associations between Australian-Indian mothers’ use of controlling feeding practices, concerns and perceptions of children’s weight and children’s picky eating. Nutr. Diet..

[24] Orun E., Erdil Z., Cetinkaya S., Tufan N., Yalcin S.S. (2012). Problematic eating behaviour in Turkish children aged 12e72 months: Characteristics of mothers and children. Central Eur. J. Public Health.

[25] Moroshko I., Brennan L. (2013). Maternal controlling feeding behaviours and child eating in preschool-aged children. Nutr. Diet..

[26] Galloway A.T., Lee Y., Birch L.L. (2003). Predictors and consequences of food neophobia and pickiness in young girls. J. Am. Diet. Assoc..

[27] Shim J.E., Kim J., Mathai R.A., Tea S.K.R. (2011). Associations of infant feeding practices and picky eating behaviors of preschool children. J. Am. Diet. Assoc..

[28] Menzel J.E., Reilly E.E., Luo T.J., Kaye W.H. (2019). Conceptualizing the role of disgust in avoidant/restrictive food intake disorder: Implications for the etiology and treatment of selective eating. Int. J. Eat. Disord..

[29] Kozioł-Kozakowska A., Piórecka B. (2013). Food neophobia its determinants and health consequences. Stand. Med. Pediatr..

[30] Frank R.A., Raudenbush B. (2004). Individual differences in approach to novelty: The case of human food neophobia. Viewing Psychology as a Whole: The Integrative Science of William N. Dember.

[31] Knaapila A., Tuorila H., Silventoinen K., Keskitalo K., Kallela M., Wessman M., Peltonen L., Cherkas L.F., Spector T.D., Perola M. (2007). Food neophobia shows heritable variation in humans. Physiol. Behav..

[32] Emmett P.M., Hays N., Taylor C.M. (2018). Antecedents of picky eating behaviour in young children. Appetite.

[33] Falcigli G.A., Couch S.C., Gribble L.S., Pabst S.M., Frank R. (2000). Food Neophobia in Childhood Affects Dietary Variety. J. Am. Diet. Assoc..

[34] Marlow C.S., Forestell C.A. (2022). The effect of parental food neophobia on children’s fruit and vegetable consumption: A serial mediation model. Appetite.

[35] Finistrella V., Gianni N., Fintini D., Menghini D., Amendola S., Donini L.M., Manco M. (2024). Neophobia, sensory experience and child’s schemata contribute to food choices. Eat. Weight. Disord..

[36] Liberati A., Altman D.G., Tetzlaff J., Mulrow C., Gøtzsche P.C., Ioannidis J.P., Clarke M., Devereaux P.J., Kleijnen J., Moher D. (2009). The PRISMA statement for reporting systematic reviews and meta-analyses of studies that evaluate healthcare interventions: Explanation and elaboration. BMJ.

[37] Higgins J., Thomas J., Chandler J., Cumpston M., Li T., Page M., Welch V. (2024). Cochrane Handbook for Systematic Reviews of Interventions, Version 6.5. The Cochrane Trainng. https://training.cochrane.org/handbook/current.

[38] Centre for Reviews and Dissemination, University of York (2009). Systematic Reviews CRD’s Guidance for Undertaking Reviews in Health Care. https://www.york.ac.uk/media/crd/Systematic_Reviews.pdf.

[39] American Psychiatric Association (2023). Diagnostic and Statistical Manual of Mental Disorders.

[40] Aromataris E., Lockwood C., Porritt K., Pilla B., Jordan Z. (2024). JBI Manual for Evidence Synthesis. JBI.

[41] Higgins J.P.T., Savović J., Page M.J., Elbers R.G., Sterne J.A.C., Higgins J.P.T., Thomas J., Chandler J., Cumpston M., Li T., Page M.J., Welch V.A. (2024). Chapter 8: Assessing risk of bias in a randomized trial [last updated October 2019]. Cochrane Handbook for Systematic Reviews of Interventions Version 6.5.

[42] Wells G.A., Shea B., O’Connell D., Pereson J., Welch V., Losos M., Tugwell P. The Newcastle-Ottawa Scale (NOS) for Assessing the Quality If Nonrandomized Studies in Meta-Analyses. https://www.ohri.ca/programs/clinical_epidemiology/oxford.asp.

[43] Stang A. (2010). Critical evaluation of the Newcastle-Ottawa scale for the assessment of the quality of nonrandomized studies in meta-analyses. Eur. J. Epidemiol..

[44] Wetherill M.S., Williams M.B., Reese J., Taniguchi T., Sisson S.B., Malek-Lasater A.D., Love C.V., Jernigan V.B.B. (2021). Methods for Assessing Willingness to Try and Vegetable Consumption among Children in Indigenous Early Childcare Settings: The FRESH Study. Nutrients.

[45] Jani R., Byrne R., Love P., Agarwal C., Peng F., Yew Y.W., Panagiotakos D., Naumovski N. (2020). The Environmental and Bitter Taste Endophenotype Determinants of Picky Eating in Australian School-Aged Children 7–12 years—A Cross-Sectional Pilot Study Protocol. Int. J. Environ. Res. Public Health.

[46] Johnson S.L., Moding K.J., Maloney K., Bellows L.L. (2018). Development of the Trying New Foods Scale: A preschooler self-assessment of willingness to try new foods. Appetite.

[47] Damsbo-Svendsen M., Frøst M.B., Olsen A. (2017). Development of novel tools to measure food neophobia in children. Appetite.

[48] Steinsbekk S., Sveen T.H., Fildes A., Llewellyn C., Wichstrøm L. (2017). Screening for pickiness—A validation study. Int. J. Behav. Nutr. Phys. Act..

[49] Hollar D., Paxton-Aiken A., Fleming P. (2013). Exploratory validation of the Fruit and Vegetable Neophobia Instrument among third- to fifth-grade students. Appetite.

[50] Rubio B., Rigal N., Boireau-Ducept N., Mallet P., Meyer T. (2008). Measuring willingness to try new foods: A self-report questionnaire for French-speaking children. Appetite.

[51] Loewen R., Pliner P. (2000). The Food Situations Questionnaire: A measure of children’s willingness to try novel foods in stimulating and non-stimulating situations. Appetite.

[52] Jones L.R., Steer C.D., Rogers I.S., Emmett P.M. (2010). Influences on child fruit and vegetable intake: Sociodemographic, parental and child factors in a longitudinal cohort study. Public Health Nutr..

[53] Pereboom J., Thijs C., Eussen S., Mommers M., Gubbels J.S. (2023). Association of picky eating around age 4 with dietary intake and weight status in early adulthood: A 14-year follow-up based on the KOALA birth cohort study. Appetite.

[54] de Andrade Previato H.D.R., Behrens J.H. (2017). Taste-related factors and food neophobia: Are they associated with nutritional status and teenagers’ food choices?. Nutrition.

[55] Białek-Dratwa A., Kowalski O. (2023). Prevalence of Feeding Problems in Children and Associated Factors-A Cross-Sectional Study among Polish Children Aged 2–7 Years. Nutrients.

[56] Diamantis D.V., Emmett P.M., Taylor C.M. (2023). Effect of being a persistent picky eater on feeding difficulties in school-aged children. Appetite.

[57] Viljakainen H.T., Figueiredo R.A.O., Rounge T.B., Weiderpass E. (2019). Picky eating—A risk factor for underweight in Finnish preadolescents. Appetite.

[58] Grulichova M., Kuruczova D., Svancara J., Pikhart H., Bienertova-Vasku J. (2022). Association of Picky Eating with Weight and Height-The European Longitudinal Study of Pregnancy and Childhood (ELSPAC-CZ). Nutrients.

[59] Zohar A.H., Pick S., Lev-Ari L., Bachner-Melman R. (2020). A longitudinal study of maternal feeding and children’s picky eating. Appetite.

[60] Chao H.C., Lu J.J., Yang C.Y., Yeh P.J., Chu S.M. (2021). Serum trace element levels and their correlation with picky eating behavior, development, and physical activity in early childhood. Nutrients.

[61] Fernandez C., McCaffery H., Miller A.L., Kaciroti N., Lumeng J.C., Pesch M.H. (2020). Trajectories of picky eating in low-income US children. Pediatrics.

[62] Sandvik P., Ek A., Somaraki M., Hammar U., Eli K., Nowicka P. (2018). Picky eating in Swedish preschoolers of different weight status: Application of two new screening cut-offs. Int. J. Behav. Nutr. Phys. Act..

[63] Steinsbekk S., Bonneville-Roussy A., Fildes A., Llewellyn C.H., Wichstrøm L. (2017). Child and parent predictors of picky eating from preschool to school age. Int. J. Behav. Nutr. Phys. Act..

[64] Chao H.C., Chang H.L. (2017). Picky Eating Behaviors Linked to Inappropriate Caregiver–Child Interaction, Caregiver Intervention, and Impaired General Development in Children. Pediatr. Neonatol..

[65] Machado B.C., Dias P., Lima V.S., Campos J., Gonçalves S. (2016). Prevalence and correlates of picky eating in preschool-aged children: A population-based study. Eat. Behav..

[66] Xue Y., Lee E., Ning K., Zheng Y., Ma D., Gao H., Yang B., Bai Y., Wang P., Zhang Y. (2015). Prevalence of picky eating behaviour in Chinese school-age children and associations with anthropometric parameters and intelligence quotient. A cross-sectional study. Appetite.

[67] Xue Y., Zhao A., Cai L., Yang B., Szeto I.M.Y., Ma D., Zhang Y., Wang P. (2015). Growth and development in Chinese pre-schoolers with picky eating behaviour: A cross-sectional study. PLoS ONE.

[68] Rodríguez-Tadeo A., Patiño-Villena B., González Martínez-La Cuesta E., Urquídez-Romero R., Ros Berruezo G. (2018). Food neophobia, Mediterranean diet adherence and acceptance of healthy foods prepared in gastronomic workshops by Spanish students. Nutr. Hosp..

[69] Rodríguez-Tadeo A., Patiño Villena B., Urquidez-Romero R., Vidaña-Gaytán M.E., Periago Caston M.J., Ros Berruezo G., González Martínez-Lacuesta E. (2015). Neofobia alimentaria: Impacto sobre los hábitos alimentarios y aceptación de alimentos saludables en usuarios de comedores escolares. Nutr. Hosp..

[70] Kozioł-Kozakowska A., Piórecka B., Schlegel-Zawadzka M. (2018). Prevalence of food neophobia in pre-school children from southern Poland and its association with eating habits, dietary intake and anthropometric parameters: A cross-sectional study. Public Health Nutr..

[71] Galloway A.T., Fiorito L., Lee Y., Birch L.L. (2005). Parental pressure, dietary patterns, and weight status among girls who are “picky eaters”. J. Am. Diet. Assoc..

[72] Sdravou K., Fotoulaki M., Emmanouilidou-Fotoulaki E., Andreuoulakis E., Makris G., Sotiriadou F., Printza A. (2021). Feeding Problems in Typically Developing Young Children, a Population-Based Study. Children.

[73] Cardona Cano S., Hoek H.W., Van Hoeken D., de Barse L.M., Jaddoe V.W.V., Verhulst F.S., Tiemetier H. (2016). Behavioral outcomes of picky eating in childhood: A prospective study in the general population. J. Child. Psychol. Psychiatry.

[74] Smith A.D., Herle M., Fildes A., Cooke L., Steinsbekk S., Llewellyn C.H. (2017). Food fussiness and food neophobia share a common etiology in early childhood. J. Child. Psychol. Psychiatry Allied Discip..

[75] Cooke L.J., Haworth C.M., Wardle J. (2007). Genetic and environmental influences on children’s food neophobia. Am. J. Clin. Nutr..

[76] Faith M.S., Heo M., Keller K.L., Pietrobelli A. (2013). Child food neophobia is heritable, associated with less compliant eating, and moderates familial resemblance for BMI. Obesity.

[77] Rahill S., Kennedy A., Walton J., McNulty B.A., Kearney J. (2018). The factors associated with food fussiness in Irish school-aged children. Public Health Nutr..

[78] Yuan W.L., Rigal N., Monnery-Patris S., Chabanet C., Forhan A., Charles M.-A., de Lauzon-Guillain B., on behalf of the EDEN mother-child cohort Study Group (2016). Early determinants of food liking among 5y-old children: A longitudinal study from the EDEN mother-child cohort. Int. J. Behav. Nutr. Phys. Act..

[79] Venkatesh S., DeJesus J.M. (2022). Can children report on their own picky eating? Similarities and differences with parent report. Appetite..

[80] Caldwell A.R., Terhorst L., Magnan K., Bogen D.L. (2023). Describing and Predicting Feeding Problems During the First 2 Years Within an Urban Pediatric Primary Care Center. Clin. Pediatr. (Phila.).

[81] Brown C.L., Perrin E.M., Peterson K.E., Herb H.E.B., Horodynski M.A., Contreras D., Miller A.L., Appugliese D.P., Ball S.C., Lumeng J.C. (2018). Association of Picky Eating with Weight Status and Dietary Quality Among Low-Income Preschoolers. Acad. Pediatr..

[82] Roßbach S., Foterek K., Schmidt I., Hilbig A., Alexy U. (2016). Food neophobia in German adolescents: Determinants and association with dietary habits. Appetite.

[83] Johnson S.L., Davies P.L., Boles R.E., Gavin W.J., Bellows L.L. (2015). Young children’s food neophobia characteristics and sensory behaviors are related to their food intake. J. Nutr..

[84] Guzek D., Głąbska D., Lange E., Jezewska-Zychowicz M. (2017). A Polish Study on the influence of food neophobia in children (10-12 years old) on the intake of vegetables and fruits. Nutrients.

[85] Kutbi H.A. (2021). Picky eating in school-aged children: Sociodemographic determinants and the associations with dietary intake. Nutrients.

[86] Taquet J., Verbeken S., Goossens L. (2024). Examining the whole plate: The role of the family context in the understanding of children’s food refusal behaviors. Eat. Behav..

[87] Chen J.L., Doong J.Y., Tu M.J., Huang S.C. (2024). Impact of Dietary Coparenting and Parenting Strategies on Picky Eating Behaviors in Young Children. Nutrients.

[88] Vaarno J., Niinikoski H., Kaljonen A., Aromaa M., Lagström H. (2015). Mothers’ restrictive eating and food neophobia and fathers’ dietary quality are associated with breast-feeding duration and introduction of solid foods: The STEPS study. Public Health Nutr..

[89] Olmer Specht I., Friis Rohde J., Julie Olsen N., Lilienthal Heitmann B. (2018). Duration of Exclusive Breastfeeding May be Related to Eating Behaviour and Dietary Intake in Obesity Prone Normal Weight Young Children. PLoS ONE.

[90] Bell L.K., Jansen E., Mallan K., Magarey A.M., Daniels L. (2018). Poor dietary patterns at 1–5 years of age are related to food neophobia and breastfeeding duration but not age of introduction to solids in a relatively advantaged sample. Eat. Behav..

[91] Wolstenholme H., Kelly C., Heary C. (2022). “Fussy eating” and feeding dynamics: School children’s perceptions, experiences, and strategies. Appetite.

[92] Chilman L.B., Meredith P.J., Southon N., Kennedy-Behr A., Frakking T., Swanepoel L., Verdonck M. (2023). A qualitative inquiry of parents of extremely picky eaters: Experiences, strategies and future directions. Appetite.

[93] Lumeng J.C., Miller A.L., Appugliese D., Rosenblum K., Kaciroti N. (2018). Picky eating, pressuring feeding, and growth in toddlers. Appetite.

[94] Moding K.J., Stifter C.A. (2016). Temperamental approach/withdrawal and food neophobia in early childhood: Concurrent and longitudinal associations. Appetite.

[95] Yalcin S., Oflu A., Akturfan M., Yalcin S.S. (2022). Characteristics of picky eater children in Turkey: A cross-sectional study. Bmc Pediatr..

[96] Norton J., Raciti M.M. (2016). Primary caregivers of young children are unaware of food neophobia and food preference development. Heal. Promot. J. Aust..

[97] Coulthard H., Palfreyman Z., Morizet D. (2016). Sensory evaluation of a novel vegetable in school age children. Appetite.

[98] Moding K.J., Stifter C.A. (2016). Stability of food neophobia from infancy through early childhood. Appetite.

[99] Howard A.J., Mallan K.M., Byrne R., Magarey A., Daniels L.A. (2012). Toddlers’ food preferences. The impact of novel food exposure, maternal preferences and food neophobia. Appetite.

[100] Tan C.C., Holub S.C. (2012). Maternal feeding practices associated with food neophobia. Appetite.

[101] Wardle J., Carnell S., Cooke L. (2005). Parental control over feeding and children’s fruit and vegetable intake: How are they related?. J. Am. Diet. Assoc..

[102] Poelman A.A.M., Cochet-Broch M., Cox D.N., Vogrig D. (2019). Vegetable Education Program Positively Affects Factors Associated with Vegetable Consumption Among Australian Primary (Elementary) Schoolchildren. J. Nutr. Educ. Behav..

[103] Cassells E.L., Magarey A.M., Daniels L.A., Mallan K.M. (2014). The influence of maternal infant feeding practices and beliefs on the expression of food neophobia in toddlers. Appetite.

[104] Cooke L.J., Wardle J., Gibson E.L., Sapochnik M., Sheiham A., Lawson M. (2003). Demographic, familial and trait predictors of fruit and vegetable consumption by pre-school children. Public Health Nutr..

[105] Russell C.G., Worsley A. (2013). Why do they like that? And can I do anything about it? The nature and correlates of parents’ attributions and self-efficacy beliefs about preschool children’s food preferences. Appetite.

[106] Jordan A.A., Appugliese D.P., Miller A.L., Lumeng J.C., Rosenblum K.L., Pesch M.H. (2019). Maternal prompting types and child vegetable intake: Exploring the moderating role of picky eating. Appetite.

[107] Cole N.C., Musaad S.M., Lee S.-Y., Donovan S.M. (2018). Home feeding environment and picky eating behavior in preschool-aged children: A prospective analysis. Eat. Behav..

[108] Jacobi C., Schmitz G., Stewart Agras W. (2008). Is picky eating an eating disorder?. Int. J. Eat. Disord..

[109] Jarman M., Ogden J., Inskip H., Lawrence W., Baird J., Cooper C., Robinson S., Barker M. (2015). How do mothers manage their preschool children’s eating habits and does this change as children grow older? A longitudinal analysis. Appetite.

[110] Tharner A., Jansen P.W., Kiefte-De Jong J.C., Moll H.A., Van Der Ende J., Jaddoe V.W.V., Hofman A., Tiemeier H., Franco O.H. (2014). Toward an operative diagnosis of fussy/picky eating: A latent profile approach in a population-based cohort. Int. J. Behav. Nutr. Phys. Act..

[111] Harris H.A., Staton S., Morawska A., Gallegos D., Oakes C., Thorpe K. (2019). A comparison of maternal feeding responses to child fussy eating in low-income food secure and food insecure households. Appetite.

[112] del Campo C., Bouzas C., Monserrat-Mesquida M., Tur J.A. (2023). Assessing food preferences and neophobias among Spanish adolescents from Castilla–La Mancha. Foods.

[113] Monnery-Patris S., Wagner S., Rigal N., Schwartz C., Chabanet C., Issanchou S., Nicklaus S. (2015). Smell differential reactivity, but not taste differential reactivity, is related to food neophobia in toddlers. Appetite.

[114] Coulthard H., Blissett J. (2009). Fruit and vegetable consumption in children and their mothers. Moderating effects of child sensory sensitivity. Appetite.

[115] Coulthard H., Thakker D. (2015). Enjoyment of Tactile Play Is Associated with Lower Food Neophobia in Preschool Children. J. Acad. Nutr. Diet..

[116] Smith A.M., Roux S., Naidoo N.T., Venter D.J.L. (2005). Food choices of tactile defensive children. Nutrition.

[117] Maratos F.A., Staples P. (2015). Attentional biases towards familiar and unfamiliar foods in children. The role of food neophobia. Appetite.

[118] Monneuse M.O., Rigal N., Frelut M.L., Hladik C.M., Simmen B., Pasquet P. (2008). Taste acuity of obese adolescents and changes in food neophobia and food preferences during a weight reduction session. Appetite.

[119] Berger P.K., Hohman E.E., Marini M.E., Savage J.S., Birch L.L. (2016). Girls’ picky eating in childhood is associated with normal weight status from ages 5 to 15 y. Am. J. Clin. Nutr..

[120] Sandvik P., Ek A., Eli K., Somaraki M., Bottai M., Nowicka P. (2019). Picky eating in an obesity intervention for preschool-aged children—What role does it plays, and does the measurement instrument matter?. Int. J. Behav. Nutr. Phys. Act..

[121] Antoniou E.E., Roefs A., Kremers S.P., Jansen A., Gubbels J.S., Sleddens E.F., Thijs C. (2016). Picky eating and child weight status development: A longitudinal study. J. Hum. Nutr. Diet..

[122] Ekstein S., Laniado D., Glick B. (2010). Does picky eating affect weight-for-length measurements in young children?. Clin. Pediatr. (Phila.).

[123] Taylor C.M., Steer C.D., Hays N.P., Emmett P.M. (2019). Growth and body composition in children who are picky eaters: A longitudinal view. Eur. J. Clin. Nutr..

[124] Brown C.L., Perrin E.M. (2020). Defining picky eating and its relationship to feeding behaviors and weight status. J. Behav. Med..

[125] Knaapila A.J., A Sandell M., Vaarno J., Hoppu U., Puolimatka T., Kaljonen A., Lagström H. (2015). Food neophobia associates with lower dietary quality and higher BMI in Finnish adults. Public Health Nutr..

[126] Finistrella V., Manco M., Ferrara A., Rustico C., Presaghi F., Morino G. (2012). Cross-sectional exploration of maternal reports of food neophobia and pickiness in preschooler-mother dyads. J. Am. Coll. Nutr..

[127] Li Z., van der Horst K., Edelson-Fries L.R., Yu K., You L., Zhang Y., Vinyes-Pares G., Wang P., Ma D., Yang X. (2017). Perceptions of food intake and weight status among parents of picky eating infants and toddlers in China: A cross-sectional study. Appetite.

[128] Jacobi C., Agras W.S., Bryson S., Hammer L.D. (2003). Behavioral validation, precursors, and concomitants of picky eating in childhood. J. Am. Acad. Child. Adolesc. Psychiatry.

[129] Perry R.A., Mallan K.M., Koo J., Mauch C.E., Daniels L.A., Magarey A.M. (2015). Food neophobia and its association with diet quality and weight in children aged 24 months: A cross-sectional study. Int. J. Behav. Nutr. Phys. Act..

[130] Costa A., Silva C., Oliveira A. (2020). Food neophobia and its association with food preferences and dietary intake of adults. Nutr. Diet..

[131] van der Horst K., Deming D.M., Lesniauskas R., Carr B.T., Reidy K.C. (2016). Picky eating: Associations with child eating characteristics and food intake. Appetite.

[132] Li P., Ren Z., Zhang J., Lan H., Szeto I.M., Wang P. (2022). Consumption of Added Sugar among Chinese Toddlers and Its Association with Picky Eating and Daily Screen Time. Nutrients.

[133] Taylor C.M., Northstone K., Wernimont S.M., Emmett P.M. (2016). Macro-and micronutrient intakes in picky eaters: A cause for concern?. Am. J. Clin. Nutr..

[134] Gan K., Tithecott C., Neilson L., Seabrook J.A., Dworatzek P. (2021). Picky Eating Is Associated with Lower Nutrient Intakes from Children’s Home-Packed School Lunches. Nutrients.

[135] Dubois L., Farmer A.P., Girard M., Peterson K. (2007). Preschool children’s eating behaviours are related to dietary adequacy and body weight. Eur. J. Clin. Nutr..

[136] Guzek D., Głąbska D., Mellová B., Zadka K., Żywczyk K., Gutkowska K. (2018). Influence of food neophobia level on fruit and vegetable intake and its association with urban area of residence and physical activity in a nationwide case-control study of polish adolescents. Nutrients.

[137] De Wild V.W., Jager G., Olsen A., Costarelli V., Boer E., Zeinstra G.G. (2018). Breast-feeding duration and child eating characteristics in relation to later vegetable intake in 2-6-year-old children in ten studies throughout Europe. Clay Miner..

[138] Mustonen S., Oerlemans P., Tuorila H. (2012). Familiarity with and affective responses to foods in 8-11-year-old children. The role of food neophobia and parental education. Appetite.

[139] Russell C.G., Worsley A. (2008). A Population-based Study of Preschoolers’ Food Neophobia and Its Associations with Food Preferences. J. Nutr. Educ. Behav..

[140] Kähkönen K., Rönkä A., Hujo M., Lyytikäinen A., Nuutinen O. (2018). Sensory-based food education in early childhood education and care, willingness to choose and eat fruit and vegetables, and the moderating role of maternal education and food neophobia. Public Health Nutr..

[141] Tuorila H., Mustonen S. (2010). Reluctant trying of an unfamiliar food induces negative affection for the food. Appetite.

[142] Tsuji M., Nakamura K., Tamai Y., Wada K., Sahashi Y., Watanabe K., Ohtsuchi S., Ando K., Nagata C. (2011). Relationship of intake of plant-based foods with 6- n -propylthiouracil sensitivity and food neophobia in Japanese preschool children. Eur. J. Clin. Nutr..

[143] Fernández A., Rodríguez D.V., Ochoa C., Pedrón C., Sánchez J. (2022). Psychological and social impact on parents of children with feeding difficulties. An. Pediatría (Engl. Ed.).

[144] Fraser K., Markides B.R., Barrett N., Laws R. (2021). Fussy eating in toddlers: A content analysis of parents’ online support seeking. Matern. Child. Nutr..

[145] Maiz E., Balluerka N. (2018). Trait anxiety and self-concept among children and adolescents with food neophobia. Food Res. Int..

[146] Blomkvist E.A.M., Helland S.H., Hillesund E.R., Øverby N.C. (2018). A cluster randomized web-based intervention trial to reduce food neophobia and promote healthy diets among one-year-old children in kindergarten: Study protocol. BMC Pediatr..

[147] Allirot X., da Quinta N., Chokupermal K., Urdaneta E. (2016). Involving children in cooking activities: A potential strategy for directing food choices toward novel foods containing vegetables. Appetite.

[148] de Wild V.W.T., de Graaf C., Jager G. (2017). Use of Different Vegetable Products to Increase Preschool-Aged Children’s Preference for and Intake of a Target Vegetable: A Randomized Controlled Trial. J. Acad. Nutr. Diet..

[149] Laureati M., Bergamaschi V., Pagliarini E. (2014). School-based intervention with children. Peer-modeling, reward and repeated exposure reduce food neophobia and increase liking of fruits and vegetables. Appetite.

[150] Reverdy C., Chesnel F., Schlich P., Köster E.P., Lange C. (2008). Effect of sensory education on willingness to taste novel food in children. Appetite.

[151] Rioux C., Lafraire J., Picard D. (2018). Visual exposure and categorization performance positively influence 3- to 6-year-old children’s willingness to taste unfamiliar vegetables. Appetite.

[152] Coulthard H., Sealy A. (2017). Play with your food! Sensory play is associated with tasting of fruits and vegetables in preschool children. Appetite.

[153] Bourne L., Bryant-Waugh R., Mandy W., Solmi F. (2023). Investigating the prevalence and risk factors of picky eating in a birth cohort study. Eat. Behav..

[154] Searle B.E., Harris H.A., Thorpe K., Jansen E. (2020). What children bring to the table: The association of temperament and child fussy eating with maternal and paternal mealtime structure. Appetite.

[155] Pickard A., Thibaut J., Philippe K., Lafraire J. (2023). Journal of Experimental Child Poor conceptual knowledge in the food domain and food rejection dispositions in 3- to 7-year-old children. J. Exp. Child. Psychol..

[156] Sarin H.V., Taba N., Fischer K., Esko T., Kanerva N., Moilanen L., Saltevo J., Joensuu A., Borodulin K., Männistö S. (2019). Food neophobia associates with poorer dietary quality, metabolic risk factors, and increased disease outcome risk in population-based cohorts in a metabolomics study. Am. J. Clin. Nutr..

[157] Brown C.L., Vander Schaaf E.B., Cohen G.M., Irby M.B., Skelton J.A. (2016). Association of Picky Eating and Food Neophobia with Weight: A Systematic Review. Child. Obes..

[158] Anania C., Perla F.M., Olivero F., Pacifico L., Chiesa C. (2018). Mediterranean diet and nonalcoholic fatty liver disease. World J. Gastroenterol..

[159] Kastorini C.-M., Milionis H.J., Esposito K., Giugliano D., Goudevenos J.A., Panagiotakos D.B. (2011). The effect of Mediterranean diet on metabolic syndrome and its components: A meta-analysis of 50 studies and 534,906 individuals. J. Am. Coll. Cardiol..

[160] Finicelli M., Squillaro T., Di Cristo F., Di Salle A., Melone M.A.B., Galderisi U., Peluso G. (2019). Metabolic syndrome, Mediterranean diet, and polyphenols: Evidence and perspectives. J. Cell Physiol..

[161] Puhl R.M., Schwartz M.B. (2003). If you are good you can have a cookie: How memories of childhood food rules link to adult eating behaviors. Eat. Behav..

[162] Karaaǧaç Y., Bellikci-Koyu E. (2023). A narrative review on food neophobia throughout the lifespan: Relationships with dietary behaviours and interventions to reduce it. Br. J. Nutr..

[163] Chilman L., Kennedy-Behr A., Frakking T., Swanepoel L., Verdonck M. (2021). Picky eating in children: A scoping review to examine its intrinsic and extrinsic features and how they relate to identification. Int. J. Environ. Res. Public Health.

